# Randomised controlled trial of the Limit of Detection of Troponin and ECG Discharge (LoDED) strategy versus usual care in adult patients with chest pain attending the emergency department: study protocol

**DOI:** 10.1136/bmjopen-2018-025339

**Published:** 2018-10-02

**Authors:** Edward Carlton, Sarah Campbell, Jenny Ingram, Rebecca Kandiyali, Hazel Taylor, Shahid Aziz, Peter Beresford, Jason Kendall, Adam Reuben, Jason Smith, Patricia Jane Vickery, Jonathan Richard Benger

**Affiliations:** 1 Emergency Department, North Bristol NHS Trust, Bristol, UK; 2 Peninsula Clinical Trials Unit, Plymouth University, Plymouth, UK; 3 Bristol Medical School (Population Health), University of Bristol, Bristol, UK; 4 Research Design Service South West, University Hospitals Bristol NHS Foundation Trust, Bristol, UK; 5 Department of Cardiology, North Bristol NHS Trust, Bristol, UK; 6 Department of Clinical Biochemistry, North Bristol NHS Trust, Bristol, UK; 7 Emergency Department, Royal Devon and Exeter Hospital NHS Foundation Trust, Exeter, UK; 8 Emergency Department, University Hospitals Plymouth NHS Trust, Plymouth, UK; 9 Faculty of Health and Life Sciences, University of the West of England, Bristol, UK

**Keywords:** myocardial infarction, ischaemic heart disease, health economics, cardiology, clinical chemistry

## Abstract

**Introduction:**

Observational data suggest a single high-sensitivity troponin blood test taken at emergency department (ED) presentation could be used to rule out major adverse cardiac events (MACE) in 10%–60% of ED patients with chest pain. This is done using an ‘undetectable’ cut-off (the Limit of Detection: LoD). We combined the LoD cut-off with ECG findings to create the LoDED strategy. We aim to establish whether the LoDED strategy works under real-life conditions, when compared with existing strategies, in a way that is cost-effective and acceptable to patients.

**Methods and analysis:**

This is a parallel-group pragmatic randomised controlled trial across UK EDs. Adults presenting to ED with suspected cardiac chest pain will be randomised 1:1. Existing rule-out strategies in current use across study centres, using serial high-sensitivity troponin testing, will be compared with the LoDED strategy. The primary outcome is successful early discharge (discharge from hospital within 4 hours of arrival) without MACE occurring within 30 days. Secondary outcomes include initial length of hospital stay; comparative costs; patient satisfaction and acceptability to patients. To detect a 9% difference between the early discharge rates (assuming an 8% rate in the standard care group) with 90% power, 594 patients need to be recruited, assuming a 95% follow-up rate.

**Ethics and dissemination:**

The study has been approved by the Frenchay Research Ethics Committee (reference 18/SW/0038). Results will be published in an international peer-reviewed journal. Lay summaries will be made available to patients.

**Trial registration number:**

ISRCTN86184521; Pre-results.

**Strengths and limitations of this study**

- The safety of the LoDED strategy, using a single high-sensitivity troponin blood test to rapidly exclude acute myocardial infarction, is supported by extensive observational research.
- This pragmatic multicentre randomised controlled parallel group trial is designed to detect a meaningful improvement in the rate of safe early emergency department discharge using the LoDED strategy, when compared with usual care.
- Sites will use a variety of usual care pathways and high-sensitivity troponin assays to enhance the generalisability of the findings.
- Although blinding of clinicians and patients will not be possible, participants will be randomised before troponin results are known to the treating clinician and those performing the outcome assessment data analysis will be blinded.
- We will undertake an integrated qualitative study to examine patient satisfaction.

## Introduction

The number of patients attending emergency departments (ED) across England and Wales continues to rise, with over 22 million attendances in 2014.^1^ Chest pain makes up nearly 10% of ED attendances and is the most common reason for emergency hospital admission.^2^ The majority of patients with chest pain have prolonged hospital stays during which they undergo testing to rule out acute myocardial infarction (AMI), yet 90% of patients are found to have a benign cause of chest pain, such as gastro-oesophageal reflux.^2^ Prolonged assessment leads to increased National Health Service (NHS) costs, patient anxiety and ED crowding.^3^

The need for prolonged assessment is driven by limitations in current diagnostic strategies. Clinicians rely on three elements to rule out AMI: patient history, ECG and blood test biomarkers. Patient history is an unreliable predictor of AMI,^4^ and few patients (14%) have an ECG that is diagnostic.^2^ Therefore, the majority of patients require biomarker testing. The current biomarker used to rule out AMI is troponin, a protein released into the blood when myocardial injury occurs. Highly sensitive troponin (hs-troponin) assays have been developed recently, meaning that very low concentrations can be measured.^5^ This has led to improved diagnostic accuracy earlier after chest pain onset.^6^ Current consensus guidelines recommend that rule-out hs-troponin testing can be undertaken using two samples taken at presentation and 1–3 hours later.^7 8^ Due to limitations in clinical and laboratory processing times, with strategies reliant on two blood tests, most patients are not discharged until at least 4 hours after ED attendance.^9^ A substantial proportion of patients could potentially be discharged much earlier with a single hs-troponin test taken at presentation to the ED.^10^

It has been proposed that a single hs-troponin test at presentation to the ED could be used to rule out AMI in 9%–60% of patients with very high diagnostic accuracy,^11–17^ using an ‘undetectable’ cut-off for ruling out AMI. This undetectable cut-off is called the Limit of Detection (LoD; lowest analyte concentration at which detection is feasible). Current data supporting the LoD cut-off are from observational studies and in patients who were not actually discharged based on hs-troponin results. Evaluating new diagnostic technologies with observational research alone has important limitations. It is possible that beneficial effects will be diluted because clinicians do not abide by their recommendations.^18^ Furthermore, unanticipated effects such as rebound overuse of resources have previously been reported and have meant that apparently safe strategies are not cost-effective.^19^ Therefore, the clinical and cost-effectiveness of the LoD cut-off remains unknown.

There are two hs-troponin assays currently approved by the National Institute for Health and Care Excellence (NICE): Roche hs-troponin T and Abbott hs-troponin I.^7^ The majority of centres within the Limit of Detection of Troponin and ECG Discharge (LoDED) study use the Roche hs-troponin T assay, reflecting current UK practice (information from manufacturer). The LoD for this assay is <5 ng/L. Some centres use the Abbott hs-troponin I assay with LoD of <2 ng/L. In addition, an emerging hs-troponin I assay (Beckman Coulter Access hs-troponin I) meets criteria to be defined as a high-sensitivity assay but is yet to be evaluated by NICE.^20^ Similarly, the LoD for this assay is <2 ng/L. Performance of all three assays at the LoD appears similar, with similar proportions of patients being eligible for safe early discharge.^15–17 21^ The LoD alone does not have the required diagnostic accuracy for clinical implementation.^13^ However, combining the LoD with ECG findings improves diagnostic accuracy (the LoDED strategy). In a meta-analysis of over 9000 patients evaluating the LoDED strategy with hs-troponin T, pooled sensitivity for 30-day major adverse cardiac events (MACE) was 98.0% (95% CI 94.7% to 99.3%), with 30% of patients eligible for early discharge.^16^ For the Abbott hs-troponin I assay, an analysis of 3155 patients showed a pooled sensitivity for MACE of 97.9% (95% CI 95.4% to 99.2%), with 25% of patients eligible for discharge.^15^ For the Beckman hs-troponin I assay, sensitivity for the rule-out of AMI has been shown to be >99%, in an analysis of 1871 patients, with 34% of patients eligible for discharge.^17^ These data demonstrate the efficacy (safety) of the LoDED strategy, yet they are all from observational cohort studies, where no patient was discharged based on their recommendations; therefore, the clinical effectiveness of the LoDED strategy across populations remains unknown.

The 2016 update to the NICE ‘chest pain of recent onset’ guidelines also supports the use of hs-troponin with a cut-off at the LoD. However, the strategy recommended by NICE requires that the LoD cut-off be combined with a risk score,^22^ such as the Thrombolysis in Myocardial Infarction (TIMI) score,^23^ rather than a normal ECG as we propose in the LoDED strategy. External validation of the NICE approach demonstrated that approximately 15% of patients will be suitable for early discharge, in contrast to approximately 30% using the LoDED strategy.^21^

The LoDED strategy is a straightforward diagnostic tool and current observational evidence suggests it is safe for implementation. The question this trial will answer is whether the LoDED strategy works when implemented in practice, with clinicians actually discharging patients early in significant numbers, without rebound increases in downstream costs and in a way that is acceptable to patients.

## Methods and analysis

### Study design and conduct

This is a pragmatic, multicentre, randomised controlled parallel group trial in adult patients presenting to the ED with suspected cardiac chest pain and who trigger the chest pain investigation pathway. Five hundred and ninety-four participants will be randomised in a 1:1 ratio to be managed according to the LoDED strategy (allowing discharge after a single hs-troponin test at presentation to ED if a participant has no new ischaemic ECG changes and the hs-troponin is below the LoD) or the usual care rule-out strategy in current clinical use at each study site. Usual care will usually include two hs-troponin blood tests taken between 1 and 6 hours after presentation and may vary between sites.

This is an open study. Participants’ study allocations will only be blinded to those performing central review of data for the assessment of outcomes and the statistician analysing the results. All patients will be consented and randomised before their initial hs-troponin results are known. Patients will be ineligible to participate in the trial once the initial hs-troponin result is known to the treating clinician in order to prevent selection bias. The decision to discharge a patient will be made after clinical assessment by the treating clinician. In the event of ongoing clinical concern, the clinician may proceed with further testing and/or admission at their discretion, and contrary to the allocated strategy. Information on protocol adherence will be collected to facilitate a per-protocol analysis as well as the primary intention-to-treat analysis.

The key objective of this study is to conduct a randomised controlled trial (RCT) of the LoDED strategy versus usual care to compare the proportion of patients successfully discharged within 4 hours of arrival, with no MACE during the following 30 days. We will also measure admission rates, hospital bed usage, length of stay, resource use and patient satisfaction, facilitating a health economic evaluation, and determine whether the LoDED strategy is acceptable to patients through qualitative interviews.

Day-to-day trial management is administered through the UKCRC-registered Peninsula Clinical Trials Unit (CTU) at Plymouth University and sponsored by North Bristol NHS Trust.

### Participant flow

**Figure 1:**
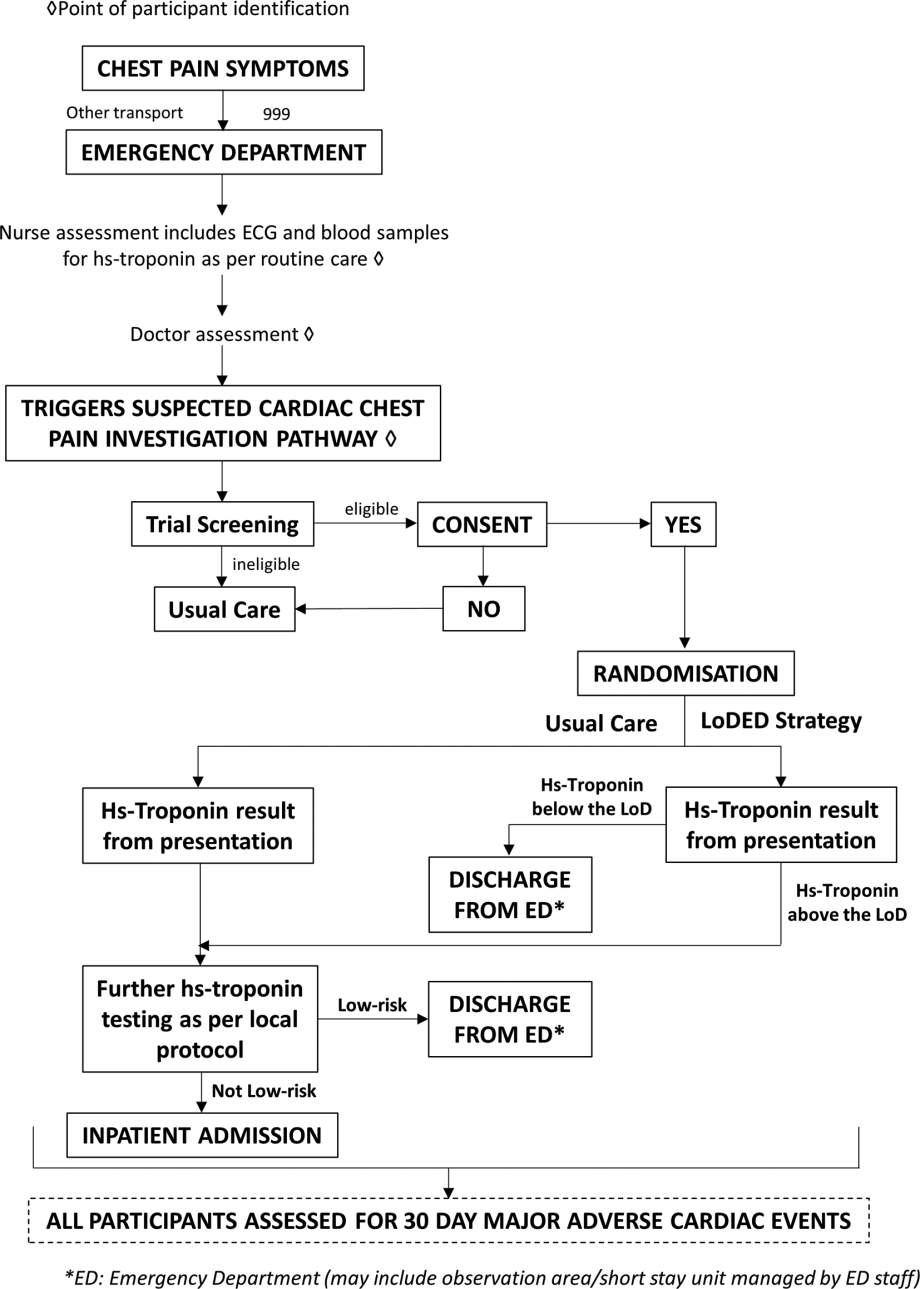
Limit of Detection of Troponin and ECG Discharge (LoDED) trial flow diagram.

Participant flow is summarised in the LoDED trial flow diagram (figure 1).

### Study population and setting

Participants will be recruited from adult (aged 18 and over) patients attending these EDs with chest pain who trigger the cardiac chest pain investigation pathway, that is, the treating clinician intends to perform investigations to rule out a cardiac cause. Full inclusion and exclusion criteria for the trial are detailed in box 1. The trial will run from 1 December 2017 until 1 December 2019. Recruitment for the trial has commenced (4 June 2018).

Box 1 **Limit of Detection of Troponin and ECG Discharge (LoDED) trial inclusion and exclusion criteria**

**Inclusion criteria**

- Age ≥18 years.
- Presenting to the emergency department (ED) with chest pain and triggering the chest pain investigation pathway, that is, the treating clinician intends to perform investigations to rule out a cardiac cause.
- Peak symptoms occurred <6 hours prior to presentation to the ED.

**Exclusion criteria**

- ST-elevation myocardial infarction or ischaemic ECG (new T wave inversion >3 mm or ST depression >1 mm) as judged by the treating clinician.
- Clear non-acute coronary syndrome (ACS) cause for chest pain found at presentation (eg, pulmonary embolism, pneumonia, aortic dissection).
- Initial hs-troponin result known to the treating clinician.
- Hospital admission indicated due to other medical/social reasons.
- Chest pain due to arrhythmia (new-onset atrial fibrillation, atrial flutter, sustained supraventricular tachycardia, second-degree or complete heart block, or sustained or recurrent ventricular arrhythmias).
- Unable to provide written informed consent (lacks capacity).
- Unwilling to provide written informed consent.
- Pain too severe to provide written informed consent.
- Follow-up will be impossible, for example, lives abroad or no fixed abode.
- Previous inclusion in the study.
- Prisoners.
- Pregnancy.
- Pre-existing renal failure requiring dialysis.

### Screening, recruitment and consent

Patient screening and recruitment, delivery of the intervention and recording of outcomes will all be carried out within participating UK NHS EDs. Potentially eligible patients will be identified at the time of arrival in the ED by clinical staff or research nurses. During triage or initial assessment the patient will be given the written study participant information sheet (PIS) by a member of the ED clinical team or research nurse. This may be before the chest pain investigation pathway has been triggered, so that a number of patients who have been given the PIS to read may subsequently be ineligible to enter the study. Due to the time taken for laboratory processing of blood samples (>60 min after presentation) and time waiting to be assessed by a doctor, it is anticipated that eligible participants will have over 1 hour to consider the PIS prior to being approached for consent.

Patients will be screened for inclusion in the study by clinical staff or research nurses, and fulfilment of initial eligibility criteria will be recorded on a study-specific screening form. The written consent process will be undertaken by an appropriately trained (Good Clinical Practice; GCP) attending clinician or an appropriate member of the research team depending on individual circumstances.

Once any questions have been answered satisfactorily, patients who are eligible and willing to participate in the study will be asked to complete and sign a consent form, which will be countersigned by the staff member receiving consent. A record of the patient’s consent to participate will be documented in the patient’s ED records, using a pre-prepared sticker. A copy of the completed consent form should be provided to the patient, a copy filed in the investigator site file and a further copy will be filed with a copy of the study PIS in the participant’s ED records.

### Clinical procedures

Study participants will have undergone the standard clinical assessment of ED patients with chest pain. This includes a triage history, routine initial observations of pulse, blood pressure, respiratory rate, oxygen saturation and the recording of a 12-lead ECG. Standard clinical practice dictates that routine blood sampling for the assessment of full blood count, urea and electrolytes and troponin is undertaken at presentation.

#### Baseline ECG

A baseline 12-lead ECG will be recorded in all patients with chest pain as part of standard clinical care. Patients identified by treating clinicians as having an ECG diagnostic of an ST-elevation myocardial infarction or evidence of new ischaemia (new T wave inversion >3 mm or ST depression >1 mm) will be ineligible.

#### Baseline blood sampling

All participants will have a blood sample taken for hs-troponin measurement on, or shortly after, ED arrival as part of the standard clinical assessment of patients with chest pain. No additional blood sampling is required for study purposes. All study centres have access to hs-troponin assays which already form part of standard care.

Given the pragmatic nature of the trial, ‘presentation’ blood sampling will be defined as the first blood sample taken after arrival in the ED. Blood sample results will not be delayed for trial purposes.

Standard laboratory reporting of hs-troponin results and other routine baseline blood tests will be used for both clinical assessment and for data collection purposes. At all sites this is through an electronic clinical record. Data from the laboratory system will be anonymously recorded within the case report form (CRF).

#### Repeat troponin tests

The default strategy will be existing rule-out strategies used in study centres (the control group). Therefore, a second hs-troponin test may be taken within these strategies so as not to delay routine clinical care. However, if allocation is to the LoDED strategy, clinicians will not be required to obtain the results of the second hs-troponin test and immediate discharge can occur.

#### Laboratory analysis

Clinical blood samples will be analysed locally in central hospital laboratories for the Elecsys hs-troponin T assay (Roche Diagnostics, 99th percentile 14 ng/L, coefficient of variation <10%, LoD 5 ng/L), Architect STAT high-sensitive troponin I (Abbott Diagnostics, 99th percentile cut-off of 26 ng/L, coefficient of variation 4%, LoD 2 ng/L) or Access hs-troponin I (Beckman Coulter, 99th percentile cut-off of 18 ng/L, coefficient of variation <10%, LoD 2 ng/L).^5 20^ Results will be made available to clinicians using laboratory reporting systems as per routine clinical care.

### Randomisation

After written consent has been obtained, participants will be randomised to be evaluated using either the existing rule-out strategy used in study centres (control) or the LoDED strategy (intervention) in a 1:1 ratio. The CTU, in conjunction with the study statistician, will provide web-based randomisation, stratified by centre and minimised by age and gender.

To randomise a participant, the recruiting doctor, ED nurse or research nurse will access the secure website and enter brief participant details (initials, date of birth, gender, study site). Once the randomisation process is complete, the computer screen will indicate which diagnostic strategy to follow, including details of the local rule-out strategy for control participants as a reminder for site staff. A printout of the allocation generated by the randomisation website will be taken and filed in the participant’s ED records.

### Trial interventions

Participants fulfilling the inclusion criteria will be randomised to be managed according to the existing local rule-out strategy (control) or the LoDED strategy (intervention).

#### Usual rule-out strategies (control)

Usual care varies between study sites but includes ECG on arrival and usually two hs-troponin tests. All sites take the first sample at presentation, but the minimum time delay between the two samples varies. The existing rule-out strategy in use at each study site will be documented at study commencement and filed in the relevant investigator site file.

#### LoDED strategy (intervention)

Participants randomised to the LoDED strategy will be eligible for discharge after a normal ECG and single hs-troponin test at presentation to the ED if the hs-troponin is below the LoD for the assay in use at the study centre.

Any participant not fulfilling this discharge criterion will revert to the existing rule-out strategy in use at that study site and have a second hs-troponin test after 1–6 hours as per usual care.

### Postintervention procedures

#### Clinical management

Once hs-troponin results are available (for either control or intervention pathways) the discharge decision will be entirely at the discretion of the treating clinician. Onward referral for outpatient investigation, such as chest pain clinics, will follow local guidance and will not be altered for trial purposes.

#### Participant advice

All participants discharged according to the LoDED strategy will be given a study-specific leaflet containing written information about the tests they have had during their stay and what to do should their chest pain returns, or if they have any concerns (online supplementary material). Feedback will be sought during the study on the content of this information leaflet and its wording refined for patient use if the LoDED strategy is adopted clinically (integrated qualitative study).

10.1136/bmjopen-2018-025339.supp1 Supplementary file 1

#### Patient satisfaction survey and EQ-5D

All participants, irrespective of group allocation, with the exception of those admitted to an inpatient ward bed for further clinical management, will be asked to complete a bespoke written patient satisfaction questionnaire on discharge from the ED (or ED observation ward; EDOU) to compare treatment satisfaction between the two study groups.^19^

Participants with an initial hs-troponin below the LoD (irrespective of group allocation) will also be asked to complete a baseline EQ-5D health status questionnaire on ED/EDOU discharge, repeated at 30 days.^24^

### Outcomes

#### Primary outcome

The primary outcome is successful early discharge, defined as discharge from hospital within 4 hours of ED arrival, without MACE occurring within 30 days.

The safety endpoint of MACE occurring within 30 days, included within the primary outcome, will be defined as cardiac death, AMI or emergency revascularisation occurring within 30 days of ED attendance (including the index presentation). AMI will be defined according to the universal definition, which states that a rise and/or fall of troponin above the 99th percentile value confirms the diagnosis.^25^ A significant rise and/or fall will be defined as an absolute change in troponin over time of at least half the 99th percentile value of the assay in clinical use.^26^

#### Secondary outcomes

Secondary outcomes will include: length of ED/EDOU stay, measured in hours, hospital admission and subsequent length of stay, incidence of MACE occurring within 30 days of ED attendance, comparative costs, participant satisfaction (quantitative survey) and acceptability to patients (qualitative methodology).

### Follow-up processes

#### Participants with an hs-troponin at presentation below the LoD

All participants with an initial hs-troponin below the LoD (irrespective of group allocation) will be followed up by telephone by the research nurse, or by email, at 30 (+5) days after index presentation to capture information about any adverse events and any primary care or secondary care health service use since discharge from the ED.

If the participant indicates that they have attended any health service provider for any reason since discharge from the ED, but there is concern over patient recall of events, then research nurses will review hospital records (where available) to verify outcomes.

#### Remaining participants

Participants with an initial hs-troponin above the LoD will be sent a screening text message after 30 (+5) days by the study team. This text will ask: ‘Since you came to the Emergency Department with chest pain have you needed to see your GP or re-attend an Emergency Department or visit an outpatient clinic for assessment of chest pain?’ Patients answering ‘No’ will require no further follow-up. Participants who fail to respond, or answer ‘Yes’, will be followed up by telephone by the research nurse or routine data will be collected from local hospital electronic patient records on initial diagnosis, local reattendance and outpatient follow-up.

#### Outcome adjudication

Incidence of MACE occurring within 30 days of ED attendance will be recorded on a serious adverse event (SAE) form by the local principal investigator or designee with reference to relevant clinical information and responses to 30-day follow-up telephone assessments uploaded to the study database. All participant data will be reviewed by an independent adjudication committee at the end of the study to confirm the primary outcome.

### Sample size

Current observational research gives estimates of the proportion of patients with an hs-troponin <LoD in the intervention group of the RCT (LoDED strategy) of between 10% and 60%.^11–17^ UK observational data suggest that these patients will be discharged within a median time of 3.5 hours (taking into account laboratory processing and 60 min of decision-making time).^10^ Data provided to us from a recent pragmatic RCT evaluating a 0/2-hour hs-troponin rule-out strategy have demonstrated that only 10% of patients will be discharged within 4 hours using this approach.^9^ Clinical protocols using rule-out strategies at later time points (eg, 3 hours) will be expected to have even fewer patients discharged within the 4-hour time frame; similarly the 0/1-hour strategy may realise a marginal increase in discharges before 4 hours. These differences have been taken into account within the power calculation. For the overall population we anticipate 8% will be discharged within 4 hours using usual care and at least 17% using the LoDED strategy. Therefore, this study will be powered to detect a 9% difference between the early discharge rates with 90% power and 5% statistical significance. This will require 282 patients in each group and 564 patients in total with primary outcome data. Assuming a 95% follow-up rate, 594 patients need to be recruited. Recruitment will take place over a 9-month period.

### Data analysis

All analyses will be conducted blind to group allocation. A Consolidated Standards of Reporting Trials diagram will be used to report the number of patients screened, recruited and randomised.^27^ It will also detail the number allocated to each group and the frequency of the primary outcome.

The primary outcome of discharge within 4 hours will be analysed by logistic regression using all those randomised in an intention-to-diagnose analysis. The analysis will be stratified by centre to allow for differences between the proportions discharged within 4 hours in the control group at each of the study sites. Heterogeneity of the odds of early discharge at each centre will be investigated and only pooled if appropriate. The OR of early discharge controlling for age, sex and centre will be presented with a 95% CI for each centre individually and combined across centres. In the event of significant heterogeneity in the control group but homogeneity within the intervention group the results will be presented as the rate of early discharge in the intervention group together with 95% CIs. Adherence to the allocated rule-out strategy will be presented as percentage with 95% CIs.

The analysis of the quantitative secondary outcomes will also control for age, sex and centre, with a multiple regression analysis and difference in means reported for the comparison of length of hospital stay between groups and for the total score from the patient satisfaction survey obtained from summing the individual items. The responses for the individual items of the patient satisfaction survey will also be reported by group, but an adjusted analysis will not be carried out.

The incidence of MACE occurring within 30 days of ED attendance in those discharged according to the LoDED strategy versus usual care will also be reported with 95% CIs. As the event rate is expected to be low, it is unlikely to be possible to carry out an adjusted analysis. There are no preplanned interim analyses. A secondary per-protocol analysis will also be performed for the primary outcome.

### Health economic analysis

The primary economic analysis will include all randomised participants (intention-to-diagnose analysis) and compare secondary care costs. Secondary care resource use will be valued using national sources of unit costs. A subgroup analysis will provide extra detail on the primary and community care costs (in addition to the secondary care costs) and consequences (quality-adjusted life-years (QALY)) for any participant with an hs-troponin below the LoD. This applies to both trial groups. Data collection for the additional primary and community care costs will be based on participant self-report following administration of a structured resource use questionnaire at 30 days and standard methods of unit costing. QALYs will be derived from the EQ-5D (5-level version) using the set of preference weights recommended by NICE at the time of analysis.^24^

### Integrated qualitative study

The qualitative component of this trial aims to explore the patients’ experiences of the ED, the concerns or anxieties they might have and how best to word patient discharge information. To explore the acceptability of the LoDED strategy to participants and inform patient discharge information resources, a qualitative research assistant will undertake semistructured interviews with a sample of intervention participants. At 30-day follow-up contact, all participants with hs-troponin levels below the LoD will be invited to participate in an interview. From those participants who consent to interview, a purposive sample will be selected across all sites to maximise variation in terms of age, sociodemographic status and gender. Up to 25 participant interviews (lasting approximately 30 min) will be conducted by phone using a topic guide to explore experiences of the participant’s stay in ED, any concerns or anxieties they might have about being discharged early and how best to word and present the written patient discharge information. Consent to take part in the qualitative study will be collected before the interview is carried out. Topic guides will be developed from the literature, input from the patient advisory group and team discussions. Two general practitioners (GP) and practice nurse focus groups will be held towards the end of the recruitment period to explore their views about the information that they would like to be provided to patients who are discharged early. Findings from the participant interviews will contribute to these focus group discussions.

Interviews and focus groups will be recorded, transcribed, anonymised and analysed using thematic methods facilitated by NVivo software (QSR International). Analysis will be ongoing and iterative. Transcripts will be coded and global themes developed from the codes. Two researchers will code a sample of transcripts independently, compare coding, discuss and resolve any discrepancies within the research team to achieve a coding consensus and ensure robust analysis.

### Safety and data monitoring

Observational data have shown the LoDED strategy has a very high diagnostic accuracy.^11–17^ However, no rule-out strategy is 100% accurate, therefore, although there is no formal data monitoring committee, adverse event data will be monitored by the Trial Steering Committee (TSC), to ensure safety. The TSC includes an independent statistician, cardiologist, emergency physician (chair) and a patient and public representative and will meet on four occasions. All suspected SAEs will be reported, within 24 hours of discovery, to the CTU who will notify the Chief Investigator (CI), the TSC and the trial sponsor. All SAEs will be followed up until resolution. If the SAE is considered a MACE, this will be indicated on the SAE form, and will be adjudicated at the end of the study. Following the initial report to the CI of any study-related SAEs, the CI will notify the chair of the TSC within 48 hours of the event. The chair will arrange an ad hoc meeting of the TSC to discuss the SAE, and agree to any actions as needed. The TSC and trial sponsor have the authority to stop the trial if any indication of harm is found.

The CTU data management team is responsible for data management, including data entry. Original CRF pages will be posted to the CTU at agreed time points for double-data entry onto an SQL Server database using a bespoke, password-protected website, designed and maintained by the CTU data programming team.

### Ethics and dissemination

The trial complies with the Declaration of Helsinki and GCP guidelines. All eligible, willing participants will undergo written informed consent by GCP trained staff before taking part in the study.

The results of the study will be applicable and of interest to emergency physicians, acute physicians, cardiologists, GPs and patients. The CI and trial management group (TMG) will establish a writing committee which will be responsible for preparing scientific reports of the study findings. The aim will be to publish a primary manuscript in a high-impact general medical journal, published as open access, with secondary analyses described in specialty journals. Primary findings will also be presented at key meetings, for example, the annual conference of the Royal College of Emergency Medicine, the European Society of Cardiology Annual Congress and the International Conference on Emergency Medicine.

### Patient and public involvement

Patient and public involvement was ensured at all stages of trial design. A prospective quantitative survey of 278 patients with chest pain, performed by our group, has previously demonstrated satisfaction with very early discharge.^28^ The intervention and primary outcome measure of safe early discharge were supported by our patient advisory group. The patient advisory group oversaw development, and approved all patient-facing materials, including PIS, consent forms and discharge information leaflets. There will be continued patient representation on the TMG, TSC and authorship. Lay summaries of trial results will be made freely available to participants and the wider public.

## Discussion

This trial of the LoDED strategy versus usual care is a pragmatic multicentre RCT aiming to establish the clinical and cost-effectiveness of a novel rule-out strategy for patients with chest pain attending the ED using just a single blood test, combined with ECG findings. Should the LoDED strategy prove effective, in a way that is acceptable to patients, it is likely to become widespread practice in those EDs with access to hs-troponin assays. This would have potential benefits to patients around the world, by providing early reassurance and a potential reduction in ED crowding by facilitating earlier discharge.

This is a pragmatic study, being carried out in an environment where patients with chest pain are normally treated, with clinical assessment being undertaken by treating emergency clinicians. In addition, we have selected multiple sites with variable usual care pathways that use a range of commercially available hs-troponin assays. These elements of the study design will enhance the generalisability of the trial findings.

The trial has also been designed to meet its objectives and recruitment targets in the challenging ED environment using a carefully considered effect size and accrual rate, based on data from previous research. We believe the trial will therefore be able to deliver these objectives within the allocated time and resources. Previous and ongoing interventional trials in this area have chosen a step-wedged cluster randomised design which require a larger sample size than individual randomisation, and have had primary outcomes evaluating safety.^18 29^ Given the strength of observational data confirming the high diagnostic accuracy of the LoDED strategy, we have been able to focus on clinical effectiveness as a primary outcome and we have elected to use a parallel group design with 1:1 randomisation. This has allowed us to maximise the efficiency of the trial.

Other rule-out strategies using a single blood test to facilitate early discharge have been considered.^10 18 21 29 30^ However, the LoDED strategy may have advantages over these. First, the hs-troponin test can be applied, on arrival in the ED, independently of chest pain onset time across the study population. This sets it apart from alternative methods currently under evaluation such as HighSTEACS,^29^ which uses a higher Abbott hs-troponin I cut-off value. The HighSTEACS strategy cannot be applied in patients presenting less than 2 hours from symptom onset, which is likely to make it less applicable and effective than the LoDED strategy. Second, other alternative strategies, including those recommended by NICE, require the addition of a risk score such as TIMI.^22^ This may act to reduce the proportion of patients suitable for early discharge when compared with the LoDED strategy.

## Conclusions

The LoDED trial will seek to determine whether the LoDED strategy works when implemented in practice, with clinicians discharging patients early in significant numbers, without rebound increases in downstream costs and in a way that is acceptable to patients. It is an important trial for patients with chest pain presenting to the ED and the clinicians involved in their care. If we demonstrate that the LoDED strategy avoids the need for prolonged assessment in an extra 9% of patients or more, in a way that is cost-effective and acceptable to patients, we anticipate widespread uptake of the strategy, leading to earlier reassurance for patients and substantial cost savings for the health system.

## Supplementary Material

Reviewer comments

Author's manuscript
