# Supplementary material for: Randomised controlled trial of the Limit of Detection of Troponin and ECG Discharge (LoDED) strategy versus usual care in adult patients with chest pain attending the emergency department: study protocol
Source: BMJ Open. 2018 Oct 2;8(10):e025339. doi: 10.1136/bmjopen-2018-025339 (PMC6169748; doi:10.1136/bmjopen-2018-025339)
Supplement: Supplementary file 1 [file bmjopen-2018-025339supp001.pdf]

Randomised controlled trial of the LoDED (Limit of Detection of Troponin and ECG Discharge) strategy versus usual care in adult chest pain patients attending the Emergency Department

Supplement: Patient discharge information leaflet (for those participants discharged according to the LoDED strategy)

AFTER DISCHARGE

If you have chest pain again that worries you or if any of your symptoms return or change, then call 999 or come back to the Emergency Department.

Following your visit, it is important to stay healthy to help your recovery. This includes stopping smoking if you smoke, reducing the amount of alcohol you drink, exercising and eating healthily. Your GP can help you with this. It's a good idea to visit them soon.

CONTACT US

Details to go here.

LoDED SAFE & RAPID CHEST PAIN MANAGEMENT

North Bristol NHS
NHS Trust

LoDED
SAFE & RAPID CHEST PAIN MANAGEMENT

Discharge Information

YOUR CHEST PAIN

Chest pain is very common and can be caused by a number of reasons, many of which are not serious. We have done some tests to see whether we think your chest pain is caused by a heart attack.

WHAT TESTS HAVE WE DONE?

- A blood test, called troponin, to check whether there are any signs of heart muscle damage in your blood.

  In your case, we have not detected any signs of heart muscle damage. This means it is very unlikely that you have had a heart attack.

- A heart trace (ECG) to check the activity of the heart and to check that the heart is getting enough oxygen.

  In your case your heart trace is normal. This means you have a healthy heart.

WHAT IS A HEART ATTACK?

A heart attack happens when there is a sudden loss of blood flow to a part of your heart muscle. Most heart attacks are caused by coronary heart disease. Coronary heart disease is when your coronary arteries (the arteries that supply your heart muscle with oxygen-rich blood) become narrowed by a gradual build-up of fatty material within their walls. Coronary heart disease can be treated using medication or a surgical procedure to restore blood flow.

YOUR PERSONAL RISK

The chance that you have had a heart attack is very low at less than 1%:

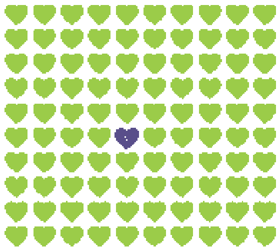

For every 100 people that come into the emergency department like you with chest pain: 99 people will not have a heart attack or any heart complications in the next 30 days; one person will have a heart attack or a heart complication in the next 30 days. Although we do not believe you have had a heart attack, we cannot rule this out entirely, we can only calculate your risk.

WHAT HAPPENS NOW?

We believe it is safe for you to go home. However, if you are anxious or concerned about this, please let your doctor know and they can discuss your options with you. We can repeat the troponin blood test after 3 hours. Doing this allows us to be more even more confident that you have not had a heart attack. You will have to wait for the results which can take up to 4 or 5 hours in total.
