# Supplementary material for: Randomised controlled trial of the Limit of Detection of Troponin and ECG Discharge (LoDED) strategy versus usual care in adult patients with chest pain attending the emergency department: study protocol
Source: BMJ Open. 2018 Oct 2;8(10):e025339. doi: 10.1136/bmjopen-2018-025339 (PMC6169748; doi:10.1136/bmjopen-2018-025339)
Supplement: Author's manuscript [file bmjopen-2018-025339.draft_revisions.pdf]

BMJ Open

BMJ Open is committed to open peer review. As part of this commitment we make the peer review history of every article we publish publicly available.

When an article is published we post the peer reviewers' comments and the authors' responses online. We also post the versions of the paper that were used during peer review. These are the versions that the peer review comments apply to.

The versions of the paper that follow are the versions that were submitted during the peer review process. They are not the versions of record or the final published versions. They should not be cited or distributed as the published version of this manuscript.

BMJ Open is an open access journal and the full, final, typeset and author-corrected version of record of the manuscript is available on our site with no access controls, subscription charges or pay-per-view fees (http://bmjopen.bmj.com).

If you have any questions on BMJ Open's open peer review process please email info.bmjopen@bmj.com

BMJ Open

Randomised controlled trial of the LoDED (Limit of Detection of Troponin and ECG Discharge) strategy versus usual care in adult chest pain patients attending the Emergency Department

Journal:	*BMJ Open*
Manuscript ID	bmjopen-2018-025339
Article Type:	Protocol
Date Submitted by the Author:	17-Jul-2018

Complete List of Authors:	Carlton, Edward; North Bristol NHS Trust, Emergency Department<br>Campbell, Sarah; Peninsula Clinical Trials Unit,<br>Ingram, Jenny; Bristol University , School of Social & Community Medicine<br>Kandiyali, Rebecca; University of Bristol School of Social and Community Medicine, Centre for Child and Adolescent Health<br>Taylor, Hazel; Research Design Service South West,<br>Aziz, Shahid; North Bristol NHS Trust, Department of Cardiology<br>Beresford, Peter; North Bristol NHS Trust, Department of Clinical Biochemistry<br>Kendall, Jason; North Bristol NHS Trust, Emergency Department<br>Reuben, Adam; Royal Devon & Exeter Hospitals NHS Foundation Trust, Emergency Medicine<br>Smith, Jason; Derriford Hospital, Emergency Department; Royal Centre for Defence Medicine (Research & Academia), Academic Department of Military Emergency Medicine<br>Vickery, Patricia; Plymouth University, Peninsula Clinical Trials Unit<br>Benger, Jonathan; The University Hospitals NHS Foundation trust, Academic Department of Emergency care; The University of the West of England, Faculty of Health & Life Sciences

Keywords:	Myocardial infarction < CARDIOLOGY, ACCIDENT & EMERGENCY MEDICINE, Ischaemic heart disease < CARDIOLOGY, HEALTH ECONOMICS, Cardiology < INTERNAL MEDICINE, Clinical chemistry < PATHOLOGY

SCHOLARONE™
Manuscripts

For peer review only - http://bmjopen.bmj.com/site/about/guidelines.xhtml

Title page

Randomised controlled trial of the LoDED (Limit of Detection of Troponin and ECG Discharge) strategy versus usual care in adult chest pain patients attending the Emergency Department

Edward Carlton,[1] Sarah Campbell,[2] Jenny Ingram,[3] Rebecca Kandiyali,[3] Hazel Taylor,[4] Shahid Aziz,[5] Peter Beresford,[6] Jason Kendall,[1] Adam Reuben,[7] Jason Smith,[8] Jane Vickery,[2] Jonathan Benger[9]

Corresponding Author: Dr Edward Carlton, eddcarlton@gmail.com +447595040112

Emergency Department, Southmead Hospital, North Bristol NHS Trust, Westbury-on-Trym, Bristol, BS10 5NB, UK

Affiliations: 1. Emergency Department, North Bristol NHS Trust, Bristol, UK. 2. Peninsula Clinical Trials Unit, Plymouth University, Plymouth, UK. 3. Bristol Medical School (Population Health), University of Bristol, Bristol, UK. 4. Research Design Service South West, University Hospitals Bristol NHS Foundation Trust, Bristol, UK. 5. Department of Cardiology, North Bristol NHS Trust, Bristol, UK. 6. Department of Clinical Biochemistry, North Bristol NHS Trust, Bristol, UK. 7. Emergency Department, Royal Devon and Exeter Hospital NHS Foundation Trust, Exeter, UK. 8. Emergency Department, University Hospitals Plymouth NHS Trust, Plymouth, UK. 9. Faculty of Health and Life Sciences, University of the West of England, Bristol, UK.

Word Count: 4958

For peer review only

ABSTRACT (Word Count 300)

Introduction

Observational data suggest a single high-sensitivity troponin blood test taken at emergency department (ED) presentation could be used to rule out major adverse cardiac events (MACE) in 10-60% of ED chest pain patients. This is done using an "undetectable" cut-off (the Limit of Detection: LoD). We combined the LoD cut-off with electrocardiogram findings to create the LoDED strategy. We aim to establish whether the LoDED strategy works under real-life conditions, when compared to existing strategies, in a way that is cost-effective and acceptable to patients.

Methods and analysis

This is a parallel-group pragmatic randomised controlled trial across UK EDs. Adults presenting to ED with suspected cardiac chest pain will be randomised 1:1. Existing rule-out strategies in current use across study centres, using serial high-sensitivity troponin testing, will be compared with the LoDED strategy.

The primary outcome is successful early discharge (discharge from hospital within 4 hours of arrival) without MACE occurring within 30 days. Secondary outcomes include initial length of hospital stay; comparative costs; patient satisfaction and acceptability to patients.

To detect a 9% difference between the early discharge rates (assuming an 8% rate in the standard care group) with 90% power, 594 patients need to be recruited, assuming a 95% follow-up rate.

Ethics and Dissemination

For peer review only

The study has been approved by the Frenchay Research Ethics Committee (Reference 18/SW/0038). Results will be published in an international peer reviewed journal. Lay summaries will be made available to patients.

Implications

If we demonstrate that the LoDED strategy avoids the need for prolonged assessment in an extra 9% of patients or more, in a way that is cost-effective and acceptable, we anticipate widespread uptake of the strategy.

Registration

The study is registered with Current Controlled Trials (ISRCTN86184521) and is eligible for support from the National Institute for Health Research Clinical Research Network.

For peer review only

Strengths and Limitations of the Study

- Few intervention studies have evaluated the clinical and cost effectiveness of very rapid strategies for the exclusion of acute myocardial infarction in adult chest pain patients attending the emergency department (ED).
- This pragmatic multi-centre randomised controlled trial is designed to detect a meaningful improvement in the rate of safe early ED discharge using a novel "rule out" strategy of a single high-sensitivity troponin blood test and electrocardiogram findings, when compared to usual care.
- Sites will use a variety of usual care pathways and high-sensitivity troponin assays to enhance the generalisability of the findings.
- Although blinding of clinicians and patients will not be possible, participants will be randomised before troponin results are known to the treating clinician and those performing the outcome assessment data analysis will be blinded.
- The acceptability of very rapid discharge in ED chest pain patients is unknown. We will therefore undertake an integrated qualitative study to examine patient satisfaction.

For peer review only

INTRODUCTION

The number of patients attending Emergency Departments (EDs) across England and Wales continues to rise, with over 22 million attendances in 2014.[1] Chest pain makes up nearly 10% of ED attendances and is the most common reason for emergency hospital admission.[2] The majority of patients with chest pain have prolonged hospital stays during which they undergo testing to rule out acute myocardial infarction (AMI), yet 90% of patients are found to have a benign cause of chest pain, such as gastro-oesophageal reflux.[2] Prolonged assessment leads to increased NHS costs, patient anxiety, and ED crowding.[3]

The need for prolonged assessment is driven by limitations in current diagnostic strategies. Clinicians rely on three elements to rule out AMI: patient history, electrocardiogram (ECG) and blood test biomarkers. Patient history is an unreliable predictor of AMI,[4] and few patients (14%) have an ECG that is diagnostic.[2] Therefore, the majority of patients require biomarker testing. The current biomarker used to rule-out AMI is troponin, a protein released into the blood when myocardial injury occurs. Highly sensitive troponin (hs-troponin) assays have been developed recently, meaning that very low concentrations can be measured.[5] This has led to improved diagnostic accuracy earlier after chest pain onset.[6] Current consensus guidelines recommend that rule-out hs-troponin testing can be undertaken using two samples taken at presentation and 1 to 3 hours later.[7,8] Due to limitations in clinical and laboratory processing times, with strategies reliant on two blood tests, most patients are not discharged until at least 4 hours after ED attendance.[9] A substantial proportion of patients could potentially be discharged much earlier with a single hs-troponin test taken at presentation to the ED.[10]

For peer review only

It has been proposed that a single hs-troponin test at presentation to the ED could be used to rule-out AMI in 9-60% of patients with very high diagnostic accuracy,[11-17] using an "undetectable" cut-off for ruling out AMI. This undetectable cut-off is called the Limit of Detection (LoD; lowest analyte concentration at which detection is feasible). Current data supporting the LoD cut-off are from observational studies and in patients who were not actually discharged based on hs-troponin results. Evaluating new diagnostic technologies with observational research alone has important limitations. It is possible that beneficial effects will be diluted because clinicians do not abide by their recommendations.[18] Furthermore, unanticipated effects such as rebound overuse of resources have previously been reported and have meant that apparently safe strategies are not cost-effective.[19] Therefore the clinical and cost-effectiveness of the LoD cut-off remains unknown.

There are two hs-troponin assays currently approved by the National Institute of Health and Care Excellence (NICE): Roche hs-troponin T and Abbott hs-troponin I.[7] The majority of centres within LoDED study use the Roche hs-troponin T assay, reflecting current UK practice (information from manufacturer). The LoD for this assay is <5ng/L. Some centres use the Abbott hs-troponin I assay with LoD of <2ng/L. In addition, an emerging hs-troponin I assay (Beckman Coulter Access hs-troponin I) meets criteria to be defined as a high-sensitivity assay but is yet to be evaluated by NICE.[20] Similarly, the LoD for this assay is <2ng/L. Performance of all three assays at the LoD appears similar, with similar proportions of patients being eligible for safe early discharge.[15-17,21] The LoD alone does not have the required diagnostic accuracy for clinical implementation.[13] However, combining the LoD with ECG findings improves diagnostic accuracy (the LoDED strategy). In a meta-analysis

For peer review only

of over 9000 patients evaluating the LoDED strategy with hs-troponin T, pooled sensitivity for 30-day major adverse cardiac events (MACE), was 98.0% (95% Confidence Interval [CI] 94.7% to 99.3%), with 30% of patients eligible for early discharge.[16] For the Abbott hs-troponin I assay, an analysis of 3155 patients showed a pooled sensitivity for MACE of 97.9% (95% CI 95.4% to 99.2%), with 25% of patients eligible for discharge.[15] For the Beckman hs-troponin I assay, sensitivity for the rule-out of AMI has been shown to be >99%, in an analysis of 1871 patients, with 34% of patients eligible for discharge.[17] These data demonstrate the efficacy (safety) of the LoDED strategy, yet they are all from observational cohort studies, where no patient was discharged based on their recommendations; therefore the clinical effectiveness of the LoDED strategy across populations remains unknown.

The 2016 update to the NICE "chest pain of recent onset" guidelines also support the use of hs-troponin with a cut-off at the LoD. However, the strategy recommended by NICE requires that the LoD cut-off be combined with a risk score,[22] such as the Thrombolysis in Myocardial Infarction (TIMI) score,[23] rather than a normal electrocardiogram (ECG) as we propose in the LoDED strategy. External validation of the NICE approach demonstrated that approximately 15% of patients will be suitable for early discharge, in contrast to approximately 30% using the LoDED strategy.[21]

The LoDED strategy is a straightforward diagnostic tool and current observational evidence suggests it is safe for implementation. The question this trial will answer is whether the LoDED strategy works when implemented in practice, with clinicians actually discharging patients early in significant numbers, without rebound increases in downstream costs and in a way that is acceptable to patients.

For peer review only

METHODS AND ANALYSIS

Study Design and Conduct

This is a pragmatic, multi-centre, randomised controlled parallel group trial in adult patients presenting to the ED with suspected cardiac chest pain and who trigger the chest pain investigation pathway. Five hundred and ninety four participants will be randomised in a 1:1 ratio to be managed according to the LoDED strategy (allowing discharge after a single hs-troponin test at presentation to ED if a participant has no new ischaemic ECG changes and the hs-troponin is below the LoD) or the usual care rule-out strategy in current clinical use at each study site. Usual care will usually include two hs-troponin blood tests taken between 1-6 hours after presentation and may vary between sites.

This is an open study. Participants' study allocations will only be blinded to those performing central review of data for the assessment of outcomes and the statistician analysing the results. All patients will be consented and randomised before their initial hs-troponin results are known. Patients will be ineligible to participate in the trial once the initial hs-troponin result is known to the treating clinician in order to prevent selection bias. The decision to discharge a patient will be made after clinical assessment by the treating clinician. In the event of ongoing clinical concern, the clinician may proceed with further testing and/or admission at their discretion, and contrary to the allocated strategy. Information on protocol adherence will be collected to facilitate a per-protocol analysis as well as the primary intention to treat analysis.

The key objective of this study is to conduct a randomised controlled trial of the LoDED strategy versus usual care to compare the proportion of patients successfully discharged within four hours of arrival, with no major adverse cardiac events (MACE) during the

For peer review only

following 30 days. We will also measure admission rates, hospital bed usage, length of stay, resource use and patient satisfaction, facilitating a health economic evaluation, and determine whether the LoDED strategy is acceptable to patients through qualitative interviews.

Patient and public involvement was ensured at all stages of trial design, and will continue through a patient advisory group and patient representation on the trial management group (TMG), trial steering committee (TSC) and authorship. Day-to-day trial management is administered through the UKCRC-registered Peninsula Clinical Trials Unit (CTU) at Plymouth University and sponsored by North Bristol NHS Trust.

Participant Flow

Participant flow is summarised in the LoDED trial flow diagram (Figure 1).

Study population and setting

Participants will be recruited from adult (aged 18 and over) patients attending these EDs with chest pain who trigger the cardiac chest pain investigation pathway, i.e. the treating clinician intends to perform investigations to rule out a cardiac cause. Full inclusion and exclusion criteria for the trial are detailed in box 1. The trial will run from December 1st 2017 until December 1st 2019. Recruitment for the trial has commenced (June 4th 2018).

For peer review only

Box 1. LoDED trial inclusion and exclusion criteria

Inclusion criteria

1. Age ≥18 years
2. Presenting to the ED with chest pain and triggering the chest pain investigation pathway, i.e. the treating clinician intends to perform investigations to rule out a cardiac cause
3. Peak symptoms occurred <6 hours prior to presentation to the ED

Exclusion criteria

1. ST-elevation myocardial infarction or ischaemic ECG (new T wave inversion >3mm or ST depression >1mm) as judged by the treating clinician
2. Clear non-ACS cause for chest pain found at presentation (e.g. pulmonary embolism, pneumonia, aortic dissection)
3. Initial hs-troponin result known to the treating clinician
4. Hospital admission indicated due to other medical/social reasons
5. Chest pain due to arrhythmia (new-onset atrial fibrillation, atrial flutter, sustained supraventricular tachycardia, second-degree or complete heart block, or sustained or recurrent ventricular arrhythmias)
6. Unable to provide written informed consent (lacks capacity)
7. Unwilling to provide written informed consent
8. Pain too severe to provide written informed consent
9. Follow-up will be impossible, e.g. lives abroad or no fixed abode
10. Previous inclusion in the study
11. Prisoners
12. Pregnancy
13. Pre-existing renal failure requiring dialysis

Screening, recruitment and consent

Patient screening and recruitment, delivery of the intervention and recording of outcomes will all be carried out within participating UK NHS EDs. Potentially eligible patients will be identified at the time of arrival in the ED by clinical staff or research nurses. During triage or initial assessment the patient will be given the written study participant information sheet (PIS) by a member of the ED clinical team or research nurse. This may be before the chest pain investigation pathway has been triggered, so that a number of patients who have been given the participant information sheet to read may subsequently be ineligible to enter the study. Due to the time taken for laboratory processing of blood samples (>60 minutes after presentation) and time waiting to be assessed by a doctor, it is anticipated that eligible participants will have over one hour to consider the PIS prior to being approached for consent.

Patients will be screened for inclusion in the study by clinical staff or research nurses, and fulfilment of initial eligibility criteria will be recorded on a study-specific screening form. The written consent process will be undertaken by an appropriately trained (Good Clinical Practice; GCP) attending clinician or an appropriate member of the research team depending on individual circumstances.

Once any questions have been answered satisfactorily, patients who are eligible and willing to participate in the study will be asked to complete and sign a Consent Form, which will be countersigned by the staff member receiving consent. A record of the patient’s consent to participate will be documented in the patient’s ED records, using a pre-prepared sticker. A

For peer review only

copy of the completed consent form should be provided to the patient, a copy filed in the investigator site file and a further copy will be filed with a copy of the study PIS in the participant's ED records.

Clinical procedures

Study participants will have undergone the standard clinical assessment of ED patients with chest pain. This includes a triage history, routine initial observations of pulse, blood pressure, respiratory rate, oxygen saturation and the recording of a 12-lead electrocardiogram (ECG). Standard clinical practice dictates that routine blood sampling for the assessment of full blood count, urea and electrolytes and troponin is undertaken at presentation.

Baseline ECG

A baseline 12-lead ECG will be recorded in all patients with chest pain as part of standard clinical care. Patients identified by treating clinicians as having an ECG diagnostic of an ST-elevation myocardial infarction or evidence of new ischaemia (new T-wave inversion >3mm or ST depression >1mm) will be ineligible.

Baseline blood sampling

All participants will have a blood sample taken for hs-troponin measurement on, or shortly after, ED arrival as part of the standard clinical assessment of patients with chest pain. No additional blood sampling is required for study purposes. All study centres have access to hs-troponin assays which already form part of standard care.

For peer review only

Given the pragmatic nature of the trial, "presentation" blood sampling will be defined as the first blood sample taken after arrival in the ED. Blood sample results will not be delayed for trial purposes.

Standard laboratory reporting of hs-troponin results and other routine baseline blood tests will be used for both clinical assessment and for data collection purposes. At all sites this is through an electronic clinical record. Data from the laboratory system will be anonymously recorded within the Case Report Form (CRF).

Repeat troponin tests

The default strategy will be existing rule-out strategies used in study centres (the control group). Therefore, a second hs-troponin test may be taken within these strategies so as not to delay routine clinical care. However, if allocation is to the LoDED strategy, clinicians will not be required to obtain the results of the second hs-troponin test and immediate discharge can occur.

Laboratory analysis

Clinical blood samples will be analysed locally in central hospital laboratories for the Elecsys hs-troponin T assay (Roche Diagnostics, 99th percentile 14ng/L, co-efficient of variation <10%, LoD 5ng/L), Architect STAT high sensitive troponin I (Abbott Diagnostics, 99th percentile cut-off of 26ng/L, co-efficient of variation 4%, LoD 2ng/L) or Access hs-troponin I (Beckman Coulter, 99th percentile cut-off of 18ng/L, co-efficient of variation <10%, LoD 2ng/L).[5,20] Results will be made available to clinicians using laboratory reporting systems as per routine clinical care.

For peer review only

Randomisation

After written consent has been obtained, participants will be randomised to be evaluated using either the existing rule-out strategy used in study centres (control) or the LoDED strategy (intervention) in a 1:1 ratio. The CTU, in conjunction with the study statistician, will provide web-based randomisation, stratified by centre and minimised by age and gender.

To randomise a participant, the recruiting doctor, ED nurse or research nurse will access the secure website and enter brief participant details (initials, date of birth, gender, study site). Once the randomisation process is complete, the computer screen will indicate which diagnostic strategy to follow, including details of the local rule-out strategy for control participants as a reminder for site staff. A print-out of the allocation generated by the randomisation website will be taken and filed in the participant's ED records.

Trial interventions

Participants fulfilling the inclusion criteria will be randomised to be managed according to the existing local rule-out strategy (control) or the LoDED strategy (intervention).

Usual rule-out strategies (control)

Usual care varies between study sites but includes ECG on arrival and usually two hs-troponin tests. All sites take the first sample at presentation, but the minimum time delay between the two samples varies. The existing rule-out strategy in use at each study site will be documented at study commencement and filed in the relevant investigator site file.

LoDED strategy (intervention)

For peer review only

Participants randomised to the LoDED strategy will be eligible for discharge after a normal ECG and single hs-troponin test at presentation to the ED if the hs-troponin is below the LoD for the assay in use at the study centre.

Any participant not fulfilling this discharge criterion will revert to the existing rule-out strategy in use at that study site and have a second hs-troponin test after 1-6 hours as per usual care.

Post intervention procedures

Clinical management

Once hs-troponin results are available (for either control or intervention pathways) the discharge decision will be entirely at the discretion of the treating clinician. Onward referral for outpatient investigation, such as chest pain clinics, will follow local guidance and will not be altered for trial purposes.

Participant advice

All participants discharged according to the LoDED strategy will be given a study-specific leaflet containing written information about the tests they have had during their stay and what to do should their chest pain returns, or if they have any concerns (Supplement). Feedback will be sought during the study on the content of this information leaflet and its wording refined for patient use if the LoDED strategy is adopted clinically (Integrated Qualitative Study).

Patient satisfaction survey and EQ-5D

All participants, irrespective of group allocation, with the exception of those admitted to an inpatient ward bed for further clinical management, will be asked to complete a bespoke

For peer review only

written patient satisfaction questionnaire upon discharge from the ED (or ED observation ward; EDOU) to compare treatment satisfaction between the two study groups.[19]

Participants with an initial hs-troponin below the LoD (irrespective of group allocation) will also be asked to complete a baseline EQ-5D health status questionnaire upon ED/EDOU discharge, repeated at 30 days.[27]

For peer review only

Outcomes

Primary outcome

The primary outcome is successful early discharge, defined as discharge from hospital within four hours of ED arrival, without MACE occurring within 30 days.

The safety endpoint of MACE occurring within 30 days, included within the primary outcome, will be defined as cardiac death, AMI or emergency revascularisation occurring within 30 days of ED attendance (including the index presentation). AMI will be defined according to the universal definition, which states that a rise and/or fall of troponin above the 99th percentile value confirms the diagnosis.[24] A significant rise and/or fall will be defined as an absolute change in troponin over time of at least half the 99th percentile value of the assay in clinical use.[25]

Secondary Outcomes

Secondary outcomes will include: length of ED/EDOU stay, measured in hours, hospital admission and subsequent length of stay, incidence of MACE occurring within 30 days of ED

attendance, comparative costs, participant satisfaction (quantitative survey), and acceptability to patients (qualitative methodology).

Follow-up processes

Participants with an hs-troponin at presentation below the LoD

All participants with an initial hs-troponin below the LoD (irrespective of group allocation) will be followed-up by telephone by the research nurse, or by e-mail, at 30 (+5) days after index presentation to capture information about any adverse events and any primary care or secondary care health service use since discharge from the ED.

If the participant indicates that they have attended any health service provider for any reason since discharge from the ED, but there is concern over patient recall of events, then research nurses will review hospital records (where available) to verify outcomes.

Remaining participants

Participants with an initial hs-troponin above the LoD will be sent a screening text message after 30 (+5) days by the study team. This text will ask: "Since you came to the Emergency Department with chest pain have you needed to see your GP or re-attend an Emergency Department or visit an outpatient clinic for assessment of chest pain?" Patients answering "No" will require no further follow-up. Participants who fail to respond, or answer "Yes", will be followed-up by telephone by the research nurse or routine data will be collected from local hospital electronic patient records on initial diagnosis, local re-attendance and outpatient follow-up.

Outcome adjudication

For peer review only

Incidence of MACE occurring within 30 days of ED attendance will be recorded on a Serious Adverse Event (SAE) form by the local PI or designee with reference to relevant clinical information and responses to 30-day follow-up telephone assessments uploaded to the study database. All participant data will be reviewed by an independent adjudication committee at the end of the study to confirm the primary outcome.

Sample size

Current observational research gives estimates of the proportion of patients with an hs-troponin <LoD in the intervention group of the RCT (LoDED strategy) of between 10% and 60%.[11-17] United Kingdom observational data suggest that these patients will be discharged within a median time of 3.5hrs (taking into account laboratory processing and 60 minutes of decision making time).[10] Data provided to us from a recent pragmatic RCT evaluating a 0/2hour hs-troponin rule-out strategy has demonstrated that only 10% of patients will be discharged within four hours using this approach.[9] Clinical protocols using rule-out strategies at later time points (e.g. three hours) will be expected to have even fewer patients discharged within the four-hour time frame; similarly the 0/1hour strategy may realise a marginal increase in discharges before four hours. These differences have been taken into account within the power calculation. For the overall population we anticipate 8% will be discharged within 4 hours using usual care and at least 17% using the LoDED strategy. Therefore, this study will be powered to detect a 9% difference between the early discharge rates with 90% power and 5% statistical significance. This will require 282 patients in each group and 564 patients in total with primary outcome data. Assuming

For peer review only

a 95% follow-up rate, 594 patients need to be recruited. Recruitment will take place over a nine month period.

Data analysis

All analysis will be conducted blind to group allocation. A CONSORT diagram will be used to report the number of patients screened, recruited and randomised.[26] It will also detail the number allocated to each group and the frequency of the primary outcome.

The primary outcome of discharge within four hours will be analysed by logistic regression using all those randomised in an intention to diagnose analysis. The analysis will be stratified by centre to allow for differences between the proportions discharged within four hours in the control group at each of the study sites. Heterogeneity of the odds of early discharge at each centre will be investigated and only pooled if appropriate. The odds ratio of early discharge controlling for age, sex and centre will be presented with a 95% confidence interval for each centre individually and combined across centres. In the event of significant heterogeneity in the control group but homogeneity within the intervention group the results will be presented as the rate of early discharge in the intervention group together with 95% confidence intervals. Adherence to the allocated rule-out strategy will be presented as percentage with 95% confidence intervals.

The analysis of the quantitative secondary outcomes will also control for age, sex and centre, with a multiple regression analysis and difference in means reported for the comparison of length of hospital stay between groups and for the total score from the patient satisfaction survey obtained from summing the individual items. The responses for

For peer review only

the individual items of the patient satisfaction survey will also be reported by group, but an adjusted analysis will not be carried out.

The incidence of MACE occurring within 30 days of ED attendance in those discharged according to the LoDED strategy vs usual care will also be reported with 95% confidence intervals. As the event rate is expected to be low, it is unlikely to be possible to carry out an adjusted analysis. There are no pre-planned interim analyses. A secondary per protocol analysis will also be performed for the primary outcome.

Health economic analysis

The primary economic analysis will include all randomised participants (intention to diagnose analysis) and compare secondary care costs. Secondary care resource use will be valued using national sources of unit costs. A subgroup analysis will provide extra detail on the primary and community care costs (in addition to the secondary care costs) and consequences (quality-adjusted life-years (QALYs)) for any participant with an hs-troponin below the LoD. This applies to both trial groups. Data collection for the additional primary and community care costs will be based on participant self-report following administration of a structured resource use questionnaire at 30 days and standard methods of unit costing. QALYs will be derived from the EQ-5D (5 level version) using the set of preference weights recommended by NICE at the time of analysis.[27]

Integrated qualitative study

For peer review only

The qualitative component of this trial aims to explore the patients' experiences of the ED, the concerns or anxieties they might have and how best to word patient discharge information. To explore the acceptability of the LoDED strategy to participants and inform patient discharge information resources, a qualitative research assistant will undertake semi-structured interviews with a sample of intervention participants. At 30-day follow-up contact, all participants with hs-troponin levels below the LoD will be invited to participate in an interview. From those participants who consent to interview, a purposive sample will be selected across all sites to maximise variation in terms of age, sociodemographic status and gender. Up to 25 participant interviews (lasting approximately 30 minutes) will be conducted by phone using a topic guide to explore experiences of the participant's stay in ED, any concerns or anxieties they might have about being discharged early and how best to word and present the written patient discharge information. Consent to take part in the qualitative study will be collected before the interview is carried out. Topic guides will be developed from the literature, input from the patient advisory group and team discussions. Two GP and practice nurse focus groups will be held towards the end of the recruitment period to explore their views about the information that they would like to be provided to patients who are discharged early. Findings from the participant interviews will contribute to these focus group discussions.

Interviews and focus groups will be recorded, transcribed, anonymised and analysed using thematic methods facilitated by NVivo software (QSR International Pty Ltd). Analysis will be ongoing and iterative. Transcripts will be coded and global themes developed from the codes. Two researchers will code a sample of transcripts independently, compare coding,

For peer review only

discuss and resolve any discrepancies within the research team to achieve a coding consensus and ensure robust analysis.

Safety and Data monitoring

Observational data have shown the LoDED strategy has a very high diagnostic accuracy.[11-17] However, no rule-out strategy is 100% accurate, therefore, although there is no formal data monitoring committee, adverse event data will be monitored by the TSC, to ensure safety. The TSC includes an independent statistician, cardiologist, emergency physician (Chair) and a patient and public representative and will meet on four occasions. All suspected SAEs will be reported, within 24 hours of discovery, to the CTU who will notify the CI, the TSC and the trial sponsor. All SAEs will be followed up until resolution. If the SAE is considered a MACE, this will be indicated on the SAE form, and will be adjudicated at the end of the study. Following the initial report to the CI of any study related SAEs, the CI will notify the Chair of the TSC within 48 hours of the event. The Chair will arrange an ad-hoc meeting of the TSC to discuss the SAE, and agree any actions as needed. The TSC and trial sponsor have the authority to stop the trial if any indication of harm is found.

The CTU data management team is responsible for data management, including data entry. Original CRF pages will be posted to the CTU at agreed time points for double-data entry on to an SQL Server database using a bespoke, password-protected website, designed and maintained by the CTU data programming team.

For peer review only

Ethics and dissemination

The trial complies with the Declaration of Helsinki and GCP guidelines. All eligible, willing participants will undergo written informed consent by GCP trained staff before taking part in the study. The study and protocol (Version 3, dated 29/05/2018) has been approved by the Frenchay Research Ethics Committee, UK (Reference 18/SW/0038).

The results of the study will be applicable and of interest to emergency physicians, acute physicians, cardiologists, general practitioners and patients. The CI and TMG will establish a writing committee which will be responsible for preparing scientific reports of the study findings. The aim will be to publish a primary manuscript in a high impact general medical journal, published as open access, with secondary analyses described in specialty journals. Primary findings will also be presented at key meetings e.g. the annual conference of the Royal College of Emergency Medicine, the European Society of Cardiology Annual Congress and the International Conference on Emergency Medicine.

DISCUSSION

This randomised controlled trial of the LoDED (Limit of Detection of Troponin and ECG Discharge) strategy versus usual care is a pragmatic multicentre randomised controlled trial aiming to establish the clinical and cost-effectiveness of a novel rule-out strategy for patients attending the ED with chest pain using just a single blood test, combined with electrocardiogram findings. Should the LoDED strategy prove effective, in a way that is acceptable to patients, it is likely to become widespread practice in those EDs with access to hs-troponin assays. This would have potential benefits to patients around

For peer review only

the world, by providing early reassurance and a potential reduction in ED crowding by facilitating earlier discharge.

This is a pragmatic study, being carried out in an environment where chest pain patients are normally treated, with clinical assessment being undertaken by treating emergency clinicians. In addition, we have selected multiple sites with variable usual care pathways that use a range of commercially available hs-troponin assays. These elements of the study design will enhance the generalisability of the trial findings.

The trial has also been designed to meet its objectives and recruitment targets in the challenging ED environment using a carefully considered effect size and accrual rate, based on data from previous research. We believe the trial will therefore be able to deliver these objectives within the allocated time and resources. Previous and ongoing interventional trials in this area have chosen a step wedged cluster randomised design which require a larger sample size than individual randomisation, and have had primary outcomes evaluating safety.[18,28] Given the strength of observational data confirming the high diagnostic accuracy of the LoDED strategy, we have been able to focus on clinical effectiveness as a primary outcome and we have elected to use a parallel group design with 1:1 randomisation. This has allowed us to maximise the efficiency of the trial.

Other rule-out strategies using a single blood test to facilitate early discharge have been considered.[10,18,21,28,29] However, the LoDED strategy may have advantages over these. Firstly, the hs-troponin test can be applied, on arrival in the ED, independently of chest pain onset time across the study population. This sets it apart from alternative methods currently under evaluation such as HighSTEACS,[28] which uses a higher Abbott hs-troponin I cut-off value. The HighSTEACS strategy cannot be applied in patients presenting less than two hours from symptom onset, which is likely to make it less applicable and effective than

For peer review only

the LoDED strategy. Secondly, other alternative strategies, including those recommended by NICE, require the addition of a risk score such as TIMI.[22] This may act to reduce the proportion of patients suitable for early discharge when compared to the LoDED strategy.

Conclusions

The LoDED trial will seek to determine whether the LoDED strategy works when implemented in practice, with clinicians discharging patients early in significant numbers, without rebound increases in downstream costs and in a way that is acceptable to patients. It is an important trial for patients presenting to the ED with chest pain and the clinicians involved in their care. If we demonstrate that the LoDED strategy avoids the need for prolonged assessment in an extra 9% of patients or more, in a way that is cost effective and acceptable to patients, we anticipate widespread uptake of the strategy, leading to earlier reassurance for patients and substantial cost savings for the health system.

For peer review only

REFERENCES

1. The Health and Social Care Information Centre. Hospital Episode Statistics, Admitted Patient Care - England, 2014-15 [cited 2015 Nov 27]. Available from: http://www.hesonline.nhs.uk.

2. Goodacre S, Cross E, Arnold J, et al. The health care burden of acute chest pain. *Heart* 2005;91:229–30.

3. Thokala P, Goodacre S, Collinson P, et al. Cost-effectiveness of presentation versus delayed troponin testing for acute myocardial infarction. *Heart* 2012;98:1498-503.

4. Carlton EW, Than M, Cullen L, Khattab A, Greaves K. 'Chest pain typicality' in suspected acute coronary syndromes and the impact of clinical experience. *Am J Med* 2015;128:1109-16.

5. Apple FS, Collinson PO. Analytical characteristics of high-sensitivity cardiac troponin assays. *Clin Chem* 2012;58:54-61.

6. Reichlin T, Hochholzer W, Bassetti S, et al. Early diagnosis of myocardial infarction with sensitive cardiac troponin assays. *N Engl J Med* 2009;361:858-67.

7. National Institute for Health and Care Excellence. Myocardial Infarction (acute): early rule out using high-sensitivity troponin tests (Elecsys Troponin T high-sensitive, ARCHITECT STAT High Sensitive Troponin-I and AccuTnI+3 assays). NICE, 2014. Available from: www.nice.org.uk/guidance/dg15.

8. Roffi M, Patrono C, Collet JP, et al. 2015 ESC Guidelines for the management of acute coronary syndromes in patients presenting without persistent ST-segment elevation: Task Force for the Management of Acute Coronary Syndromes in Patients Presenting without Persistent ST-Segment Elevation of the European Society of Cardiology (ESC). *Eur Heart J* 2016;37:267-315.

9. Than MP, Pickering JW, Aldous SJ, et al. Effectiveness of EDACS Versus ADAPT Accelerated Diagnostic Pathways for Chest Pain: A Pragmatic Randomized Controlled Trial Embedded Within Practice. *Ann Emerg Med* 2016;68:93-102.

10. Carlton EW, Cullen L, Than M, et al. A novel diagnostic protocol to identify patients suitable for discharge after a single high-sensitivity troponin. *Heart* 2015;101:1041-6.

11. Body R, Carley S, McDowell G, et al. Rapid exclusion of acute myocardial infarction in patients with undetectable troponin using a high-sensitivity assay. *J Am Coll Cardiol* 2011;58:1332-9.

12. Bandstein N, Ljung R, Johansson M, et al. Undetectable High-Sensitivity Cardiac Troponin T Level in the Emergency Department and Risk of Myocardial Infarction. *J Am Coll Cardiol* 2014;63:2569–78.

13. Zhelev Z, Hyde C, Youngman E, et al. Diagnostic accuracy of single baseline measurement of Elecsys Troponin T high-sensitive assay for diagnosis of acute myocardial infarction in emergency department: systematic review and meta-analysis. *BMJ* 2015;350:h15.

14. Body R, Burrows G, Carley S, et al. High-sensitivity cardiac troponin t concentrations below the limit of detection to exclude acute myocardial infarction: a prospective evaluation. *Clin Chem* 2015; 61:983-89.

For peer review only

15. Carlton EW, Greenslade J, Cullen L, et al. Low concentrations of high-sensitivity cardiac troponin I at presentation in the evaluation of emergency department patients with suspected acute coronary syndrome. *JAMA Cardiol* 2016;1:405-12.

16. Pickering JW, Than MP, Cullen L, et al. Rapid Rule-out of Acute Myocardial Infarction With a Single High-Sensitivity Cardiac Troponin T Measurement Below the Limit of Detection: A Collaborative Meta-analysis. *Ann Intern Med* 2017; 166:715-24.

17. Greenslade J, Cho E, Van Hise C, et al. Evaluating Rapid Rule-out of Acute Myocardial Infarction by Use of a High-Sensitivity Cardiac Troponin I Assay at Presentation. *Clin Chem* 2018;64:820-29.

18. Poldervaart JM, Reitsma JB, Backus BE, et al. Effect of Using the HEART Score in Patients With Chest Pain in the Emergency Department: A Stepped-Wedge, Cluster Randomized Trial. *Ann Intern Med* 2017; 166:689-97.

19. Fitzgerald P, Goodacre SW, Cross E, Dixon S. Cost-effectiveness of point-of-care biomarker assessment for suspected myocardial infarction: the randomized assessment of treatment using panel Assay of cardiac markers (RATPAC) trial. *Acad Emerg Med* 2011;18:488-95.

20. Pretorius CJ, Tate JR, Wilgen U, Cullen L and Ungerer JPJ. A critical evaluation of the Beckman Coulter Access hsTnI: Analytical performance, reference interval and concordance. *Clin Biochem* 2018; 55:49-55.

21. Carlton E, Pickering JW, Greenslade J, et al. Assessment of the 2016 National Institute for Health and Care Excellence high-sensitivity troponin rule-out strategy. *Heart* 2017; 104:665-72.

22. NICE guidance. Chest Pain of Recent Onset: Assessment and diagnosis (update). CG95. London: National Institute for Health and Care Excellence, 2016 (available at https://www.nice.org.uk/guidance/cg95).

23. Antman EM, Cohen M, Bernink PJ, et al. The TIMI risk score for unstable angina/non-ST elevation MI: a method for prognostication and therapeutic decision making. *JAMA* 2000;284: 835-42.

24. Thygesen K, Alpert J, Jaffe A, et al. Third universal definition of myocardial infarction. *J Am Coll Cardiol* 2012;60:1581-98.

For peer review only

25. Reichlin T, Irfan A, Twerenbold R, et al. Utility of absolute and relative changes in cardiac troponin concentrations in the early diagnosis of acute myocardial infarction. *Circulation* 2011;123:136-45.

26. http://www.consort-statement.org/ (accessed 10/04/2018).

27. National Institute for Health and Care Excellence. Position Statement on use of the EQ-5D-5L valuation set. 2017 (available at https://www.nice.org.uk/Media/Default/About/what-we-do/NICE-guidance/NICE-technology-appraisal-guidance/eq5d5l_nice_position_statement.pdf).

28. Chapman AR, Anand A, Boeddinghaus J, et al. Comparison of the Efficacy and Safety of Early Rule-Out Pathways for Acute Myocardial Infarction. *Circulation* 2017;135:1586-96.

29. Body R, Carlton E, Sperrin M, et al. Troponin-only Manchester Acute Coronary Syndromes (T-MACS) decision aid: single biomarker re-derivation and external validation in three cohorts. *Emerg Med J* 2017; 34:349-56.

Acknowledgements

This paper presents independent research funded by the National Institute for Health Research (NIHR) under its Research for Patient Benefit (RfPB) Programme (Grant Reference No. PB-PG-0416-20012). The views expressed are those of the authors and not necessarily those of the NHS, the NIHR or the Department of Health.

The authors would like to acknowledge and thank Rebecca Chapman, Peninsula Clinical Trials Unit, for all her hard work and administrative skills in support of this project and Gareth Williams, our lead patient representative, for his help and advice in the ethics application and his views on the protocol and research plans. We would also like to thank the British Heart Foundation for their help in supporting the Trial Steering Committee. In

For peer review only

addition we would like to thank John Pickering in providing data to inform our sample size calculation.

Contributors

Edward Carlton (EC) conceived the idea for the study. EC, Jason Kendall (JK), Adam Reuben (AR), Jason Smith (JS), and Jonathan Benger (JB) were responsible for the initial study design, which was refined with the help of Rebecca Kandiyali (RK), Jenny Ingram (JI), Hazel Taylor (HT) and Jane Vickery (JV) and support of our regional National Institute for Health Research (NIHR) Research Design Service. Expert advice on Cardiological and Biochemical elements of the study design was provided by Shahid Aziz (SA) and Peter Beresford (PB), respectively. HT provided the statistical plan, RK lead the design of the health economics analysis and JI developed the integrated qualitative study. JI supported the public and patient views in the study development. Sarah Campbell (SC) coordinated study set-up and finalisation of the study protocol as Trial Manager. All authors contributed to the final study design and protocol development, critically revised successive drafts of the manuscript and approved the final version. The trial management group is responsible for the conduct of the study, overseen by a Trial Steering Committee.

Funding

This research project is funded by the National Institute for Health Research (NIHR) through its Research for Patient Benefit (RfPB) Programme (Grant Reference Number PB-PG-0416-20012).

Competing interests

EC has received funding from Abbott Diagnostics for related research and honoraria for education from Astra Zeneca and Roche. There are no other competing interests to declare.

For peer review only

Ethics approval

The study has been approved by the South West Frenchay Research Ethics Committee (REC reference 18/SW/0038) and each of the participating NHS Trust's Research and Development department.

For peer review only

Figure 1. LoDED trial flow diagram

For peer review only

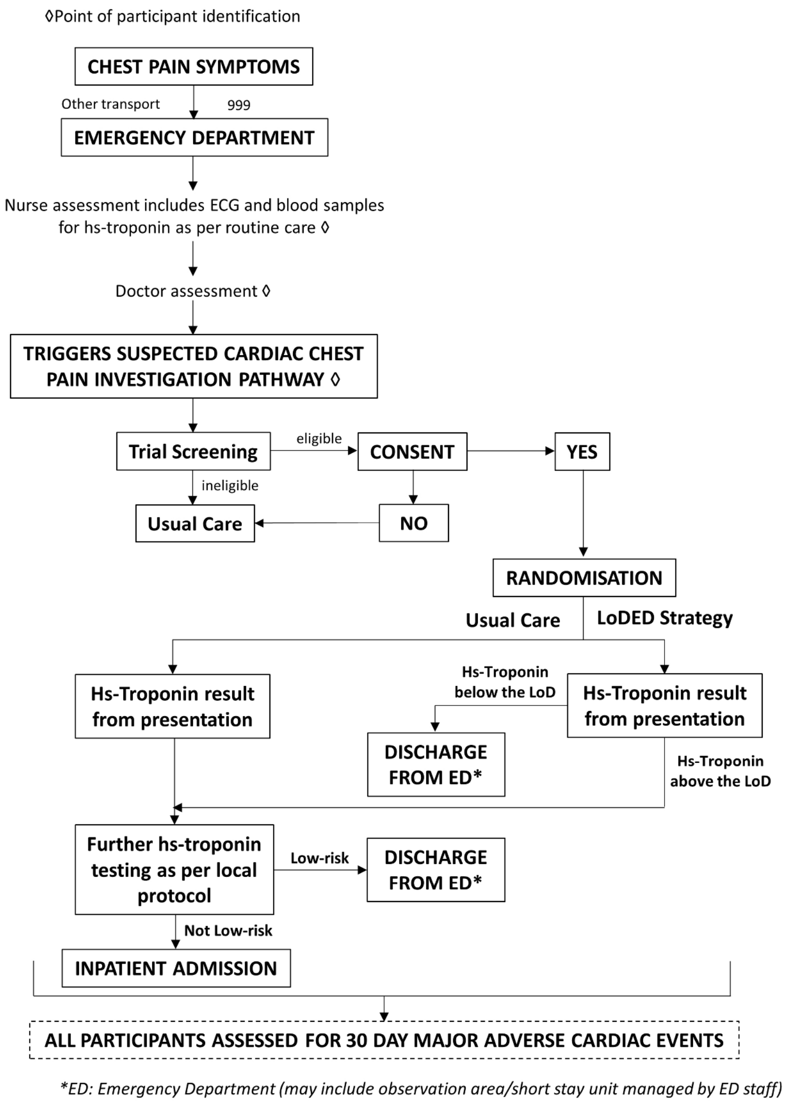

Figure 1. LoDED trial flow diagram

190x275mm (300 x 300 DPI)

Randomised controlled trial of the LoDED (Limit of Detection of Troponin and ECG Discharge) strategy versus usual care in adult chest pain patients attending the Emergency Department

Supplement: Patient discharge information leaflet (for those participants discharged according to the LoDED strategy)

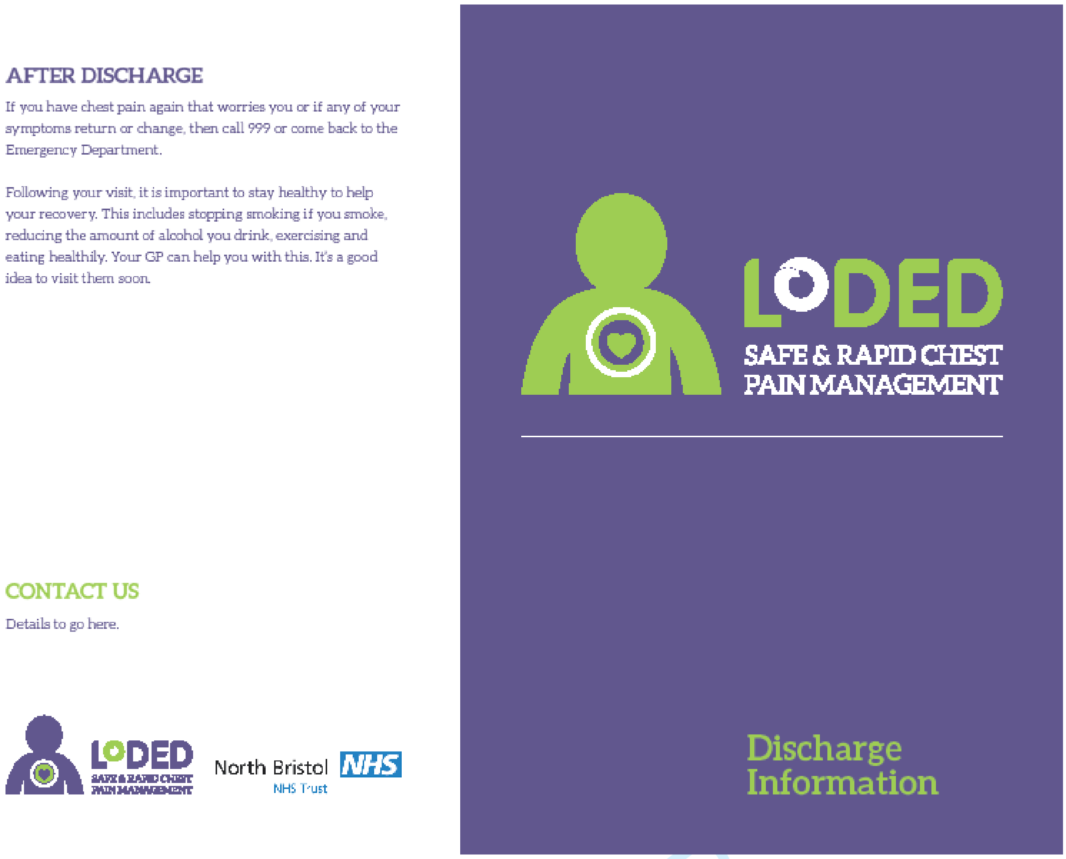
AFTER DISCHARGE

If you have chest pain again that worries you or if any of your symptoms return or change, then call 999 or come back to the Emergency Department.

Following your visit, it is important to stay healthy to help your recovery. This includes stopping smoking if you smoke, reducing the amount of alcohol you drink, exercising and eating healthily. Your GP can help you with this. It's a good idea to visit them soon.

CONTACT US

Details to go here.

LoDED SAFE & RAPID CHEST PAIN MANAGEMENT

North Bristol NHS Trust

LoDED SAFE & RAPID CHEST PAIN MANAGEMENT

Discharge Information

YOUR CHEST PAIN

Chest pain is very common and can be caused by a number of reasons, many of which are not serious. We have done some tests to see whether we think your chest pain is caused by a heart attack.

WHAT TESTS HAVE WE DONE?

- A blood test, called troponin, to check whether there are any signs of heart muscle damage in your blood.

  In your case, we have not detected any signs of heart muscle damage. This means it is very unlikely that you have had a heart attack.

- A heart trace (ECG) to check the activity of the heart and to check that the heart is getting enough oxygen.

  In your case your heart trace is normal. This means you have a healthy heart.

WHAT IS A HEART ATTACK?

A heart attack happens when there is a sudden loss of blood flow to a part of your heart muscle. Most heart attacks are caused by coronary heart disease. Coronary heart disease is when your coronary arteries (the arteries that supply your heart muscle with oxygen-rich blood) become narrowed by a gradual build-up of fatty material within their walls. Coronary heart disease can be treated using medication or a surgical procedure to restore blood flow.

YOUR PERSONAL RISK

The chance that you have had a heart attack is very low at less than 1%:

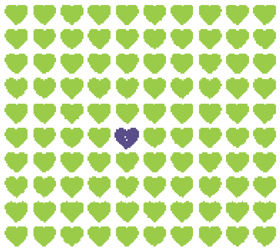

For every 100 people that come into the emergency department like you with chest pain: 99 people will not have a heart attack or any heart complications in the next 30 days; one person will have a heart attack or a heart complication in the next 30 days. Although we do not believe you have had a heart attack, we cannot rule this out entirely, we can only calculate your risk.

WHAT HAPPENS NOW?

We believe it is safe for you to go home. However, if you are anxious or concerned about this, please let your doctor know and they can discuss your options with you. We can repeat the troponin blood test after 3 hours. Doing this allows us to be more even more confident that you have not had a heart attack. You will have to wait for the results which can take up to 4 or 5 hours in total.

SPIRIT

STANDARD PROTOCOL ITEMS: RECOMMENDATIONS FOR INTERVENTIONAL TRIALS

SPIRIT 2013 Checklist: Recommended items to address in a clinical trial protocol and related documents*

Section/item	Item No	Description	Addressed on page number
**Administrative information**			
Title	1	Descriptive title identifying the study design, population, interventions, and, if applicable, trial acronym	________1____
Trial registration	2a	Trial identifier and registry name. If not yet registered, name of intended registry	________3____
	2b	All items from the World Health Organization Trial Registration Data Set	_______N/A___
Protocol version	3	Date and version identifier	_______22____
Funding	4	Sources and types of financial, material, and other support	_______28____
Roles and responsibilities	5a	Names, affiliations, and roles of protocol contributors	________1____
	5b	Name and contact information for the trial sponsor	________9____
	5c	Role of study sponsor and funders, if any, in study design; collection, management, analysis, and interpretation of data; writing of the report; and the decision to submit the report for publication, including whether they will have ultimate authority over any of these activities	________28____
	5d	Composition, roles, and responsibilities of the coordinating centre, steering committee, endpoint adjudication committee, data management team, and other individuals or groups overseeing the trial, if applicable (see Item 21a for data monitoring committee)	_________21____

For peer review only - http://bmjopen.bmj.com/site/about/guidelines.xhtml

**Introduction**			
Background and rationale	6a	Description of research question and justification for undertaking the trial, including summary of relevant studies (published and unpublished) examining benefits and harms for each intervention	_____5-7_____
	6b	Explanation for choice of comparators	______6______
Objectives	7	Specific objectives or hypotheses	________8_____
Trial design	8	Description of trial design including type of trial (eg, parallel group, crossover, factorial, single group), allocation ratio, and framework (eg, superiority, equivalence, noninferiority, exploratory)	________8_____
**Methods: Participants, interventions, and outcomes**			
Study setting	9	Description of study settings (eg, community clinic, academic hospital) and list of countries where data will be collected. Reference to where list of study sites can be obtained	_________9____
Eligibility criteria	10	Inclusion and exclusion criteria for participants. If applicable, eligibility criteria for study centres and individuals who will perform the interventions (eg, surgeons, psychotherapists)	________10____
Interventions	11a	Interventions for each group with sufficient detail to allow replication, including how and when they will be administered	________14____
	11b	Criteria for discontinuing or modifying allocated interventions for a given trial participant (eg, drug dose change in response to harms, participant request, or improving/worsening disease)	________19____
	11c	Strategies to improve adherence to intervention protocols, and any procedures for monitoring adherence (eg, drug tablet return, laboratory tests)	______19_____
	11d	Relevant concomitant care and interventions that are permitted or prohibited during the trial	________12____
Outcomes	12	Primary, secondary, and other outcomes, including the specific measurement variable (eg, systolic blood pressure), analysis metric (eg, change from baseline, final value, time to event), method of aggregation (eg, median, proportion), and time point for each outcome. Explanation of the clinical relevance of chosen efficacy and harm outcomes is strongly recommended	________16____
Participant timeline	13	Time schedule of enrolment, interventions (including any run-ins and washouts), assessments, and visits for participants. A schematic diagram is highly recommended (see Figure)	______Figure 1__

For peer review only - http://bmjopen.bmj.com/site/about/guidelines.xhtml

Sample size	14	Estimated number of participants needed to achieve study objectives and how it was determined, including clinical and statistical assumptions supporting any sample size calculations	______18_____
Recruitment	15	Strategies for achieving adequate participant enrolment to reach target sample size	______18_____

Methods: Assignment of interventions (for controlled trials)

Allocation:			
Sequence generation	16a	Method of generating the allocation sequence (eg, computer-generated random numbers), and list of any factors for stratification. To reduce predictability of a random sequence, details of any planned restriction (eg, blocking) should be provided in a separate document that is unavailable to those who enrol participants or assign interventions	______14_____
Allocation concealment mechanism	16b	Mechanism of implementing the allocation sequence (eg, central telephone; sequentially numbered, opaque, sealed envelopes), describing any steps to conceal the sequence until interventions are assigned	_______14____
Implementation	16c	Who will generate the allocation sequence, who will enrol participants, and who will assign participants to interventions	______14_____
Blinding (masking)	17a	Who will be blinded after assignment to interventions (eg, trial participants, care providers, outcome assessors, data analysts), and how	______8_____
	17b	If blinded, circumstances under which unblinding is permissible, and procedure for revealing a participant's allocated intervention during the trial	_______N/A____

Methods: Data collection, management, and analysis

Data collection methods	18a	Plans for assessment and collection of outcome, baseline, and other trial data, including any related processes to promote data quality (eg, duplicate measurements, training of assessors) and a description of study instruments (eg, questionnaires, laboratory tests) along with their reliability and validity, if known. Reference to where data collection forms can be found, if not in the protocol	_____15______
	18b	Plans to promote participant retention and complete follow-up, including list of any outcome data to be collected for participants who discontinue or deviate from intervention protocols	______15,19____

For peer review only - http://bmjopen.bmj.com/site/about/guidelines.xhtml

Data management	19	Plans for data entry, coding, security, and storage, including any related processes to promote data quality (eg, double data entry; range checks for data values). Reference to where details of data management procedures can be found, if not in the protocol	______21_____
Statistical methods	20a	Statistical methods for analysing primary and secondary outcomes. Reference to where other details of the statistical analysis plan can be found, if not in the protocol	_____18______
	20b	Methods for any additional analyses (eg, subgroup and adjusted analyses)	______18_____
	20c	Definition of analysis population relating to protocol non-adherence (eg, as randomised analysis), and any statistical methods to handle missing data (eg, multiple imputation)	______18_____
**Methods: Monitoring**			
Data monitoring	21a	Composition of data monitoring committee (DMC); summary of its role and reporting structure; statement of whether it is independent from the sponsor and competing interests; and reference to where further details about its charter can be found, if not in the protocol. Alternatively, an explanation of why a DMC is not needed	_______21______
	21b	Description of any interim analyses and stopping guidelines, including who will have access to these interim results and make the final decision to terminate the trial	______19_______
Harms	22	Plans for collecting, assessing, reporting, and managing solicited and spontaneously reported adverse events and other unintended effects of trial interventions or trial conduct	_______21_____
Auditing	23	Frequency and procedures for auditing trial conduct, if any, and whether the process will be independent from investigators and the sponsor	______21_____
**Ethics and dissemination**			
Research ethics approval	24	Plans for seeking research ethics committee/institutional review board (REC/IRB) approval	______22_____
Protocol amendments	25	Plans for communicating important protocol modifications (eg, changes to eligibility criteria, outcomes, analyses) to relevant parties (eg, investigators, REC/IRBs, trial participants, trial registries, journals, regulators)	_______22_____

For peer review only - http://bmjopen.bmj.com/site/about/guidelines.xhtml

Consent or assent	26a	Who will obtain informed consent or assent from potential trial participants or authorised surrogates, and how (see Item 32)	_______11______
	26b	Additional consent provisions for collection and use of participant data and biological specimens in ancillary studies, if applicable	_________N/A___
Confidentiality	27	How personal information about potential and enrolled participants will be collected, shared, and maintained in order to protect confidentiality before, during, and after the trial	________11/22___
Declaration of interests	28	Financial and other competing interests for principal investigators for the overall trial and each study site	________29____
Access to data	29	Statement of who will have access to the final trial dataset, and disclosure of contractual agreements that limit such access for investigators	_______22______
Ancillary and post-trial care	30	Provisions, if any, for ancillary and post-trial care, and for compensation to those who suffer harm from trial participation	______N/A_______
Dissemination policy	31a	Plans for investigators and sponsor to communicate trial results to participants, healthcare professionals, the public, and other relevant groups (eg, via publication, reporting in results databases, or other data sharing arrangements), including any publication restrictions	________22______
	31b	Authorship eligibility guidelines and any intended use of professional writers	_________22___
	31c	Plans, if any, for granting public access to the full protocol, participant-level dataset, and statistical code	_________N/A___
**Appendices**			
Informed consent materials	32	Model consent form and other related documentation given to participants and authorised surrogates	______supplied_
Biological specimens	33	Plans for collection, laboratory evaluation, and storage of biological specimens for genetic or molecular analysis in the current trial and for future use in ancillary studies, if applicable	________N/A_____

*It is strongly recommended that this checklist be read in conjunction with the SPIRIT 2013 Explanation & Elaboration for important clarification on the items. Amendments to the protocol should be tracked and dated. The SPIRIT checklist is copyrighted by the SPIRIT Group under the Creative Commons "Attribution-NonCommercial-NoDerivs 3.0 Unported" license.

For peer review only - http://bmjopen.bmj.com/site/about/guidelines.xhtml

BMJ Open

Randomised controlled trial of the LoDED (Limit of Detection of Troponin and ECG Discharge) strategy versus usual care in adult chest pain patients attending the Emergency Department: study protocol

Journal:	*BMJ Open*
Manuscript ID	bmjopen-2018-025339.R1
Article Type:	Protocol
Date Submitted by the Author:	03-Aug-2018
Complete List of Authors:	Carlton, Edward; North Bristol NHS Trust, Emergency Department<br>Campbell, Sarah; Peninsula Clinical Trials Unit,<br>Ingram, Jenny; Bristol University , School of Social & Community Medicine<br>Kandiyali, Rebecca; University of Bristol School of Social and Community Medicine, Centre for Child and Adolescent Health<br>Taylor, Hazel; Research Design Service South West,<br>Aziz, Shahid; North Bristol NHS Trust, Department of Cardiology<br>Beresford, Peter; North Bristol NHS Trust, Department of Clinical Biochemistry<br>Kendall, Jason; North Bristol NHS Trust, Emergency Department<br>Reuben, Adam; Royal Devon & Exeter Hospitals NHS Foundation Trust, Emergency Medicine<br>Smith, Jason; Derriford Hospital, Emergency Department; Royal Centre for Defence Medicine (Research & Academia), Academic Department of Military Emergency Medicine<br>Vickery, Patricia; Plymouth University, Peninsula Clinical Trials Unit<br>Benger, Jonathan; The University Hospitals NHS Foundation trust, Academic Department of Emergency care; The University of the West of England, Faculty of Health & Life Sciences
**Primary Subject Heading**:	Emergency medicine
Secondary Subject Heading:	Cardiovascular medicine, Diagnostics, Health economics, Qualitative research, Research methods
Keywords:	Myocardial infarction < CARDIOLOGY, ACCIDENT & EMERGENCY MEDICINE, Ischaemic heart disease < CARDIOLOGY, HEALTH ECONOMICS, Cardiology < INTERNAL MEDICINE, Clinical chemistry < PATHOLOGY

Note: The following files were submitted by the author for peer review, but cannot be converted to PDF. You must view these files (e.g. movies) online.

Protocol_paper_V3_Final....docx

For peer review only

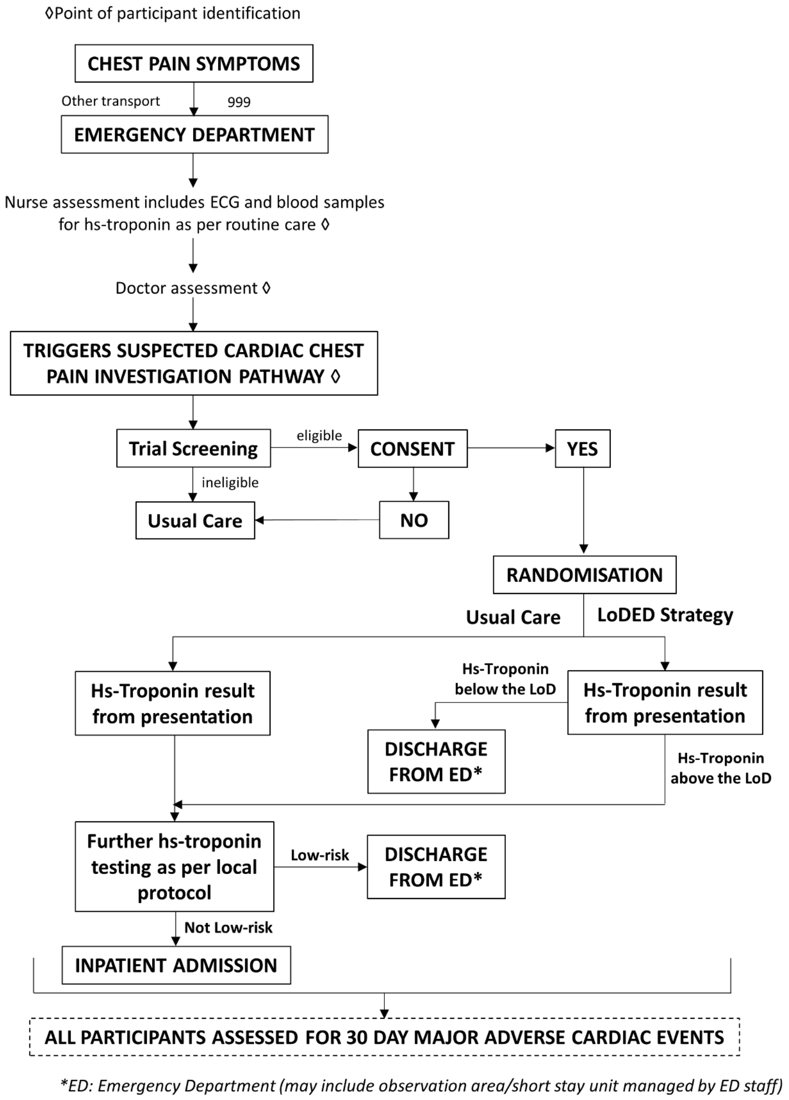

Patient flow diagram

190x275mm (300 x 300 DPI)

Randomised controlled trial of the LoDED (Limit of Detection of Troponin and ECG Discharge) strategy versus usual care in adult chest pain patients attending the Emergency Department

Supplement: Patient discharge information leaflet (for those participants discharged according to the LoDED strategy)

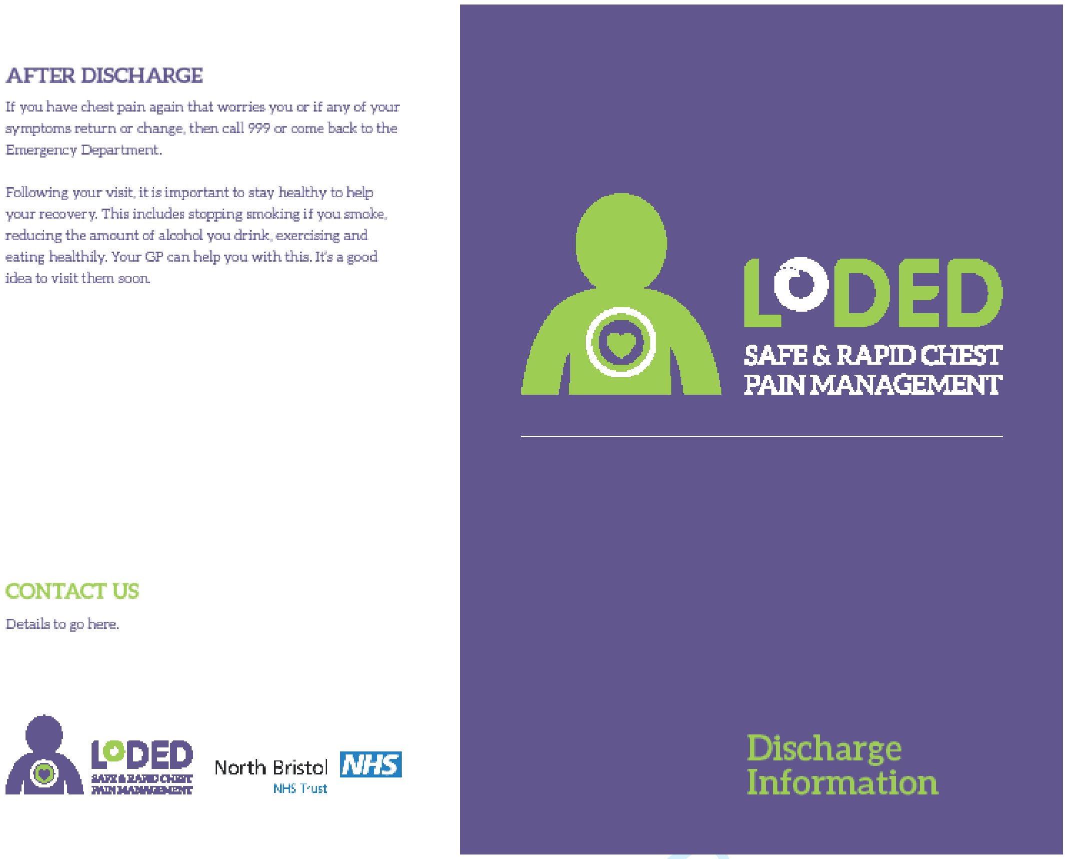

YOUR CHEST PAIN

Chest pain is very common and can be caused by a number of reasons, many of which are not serious. We have done some tests to see whether we think your chest pain is caused by a heart attack.

WHAT TESTS HAVE WE DONE?

- A blood test, called troponin, to check whether there are any signs of heart muscle damage in your blood.

  In your case, we have not detected any signs of heart muscle damage. This means it is very unlikely that you have had a heart attack.

- A heart trace (ECG) to check the activity of the heart and to check that the heart is getting enough oxygen.

  In your case your heart trace is normal. This means you have a healthy heart.

WHAT IS A HEART ATTACK?

A heart attack happens when there is a sudden loss of blood flow to a part of your heart muscle. Most heart attacks are caused by coronary heart disease. Coronary heart disease is when your coronary arteries (the arteries that supply your heart muscle with oxygen-rich blood) become narrowed by a gradual build-up of fatty material within their walls. Coronary heart disease can be treated using medication or a surgical procedure to restore blood flow.

YOUR PERSONAL RISK

The chance that you have had a heart attack is very low at less than 1%:

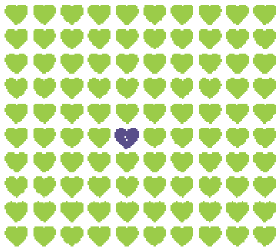

For every 100 people that come into the emergency department like you with chest pain: 99 people will not have a heart attack or any heart complications in the next 30 days; one person will have a heart attack or a heart complication in the next 30 days. Although we do not believe you have had a heart attack, we cannot rule this out entirely, we can only calculate your risk.

WHAT HAPPENS NOW?

We believe it is safe for you to go home. However, if you are anxious or concerned about this, please let your doctor know and they can discuss your options with you. We can repeat the troponin blood test after 3 hours. Doing this allows us to be more even more confident that you have not had a heart attack. You will have to wait for the results which can take up to 4 or 5 hours in total.

For peer review only

SPIRIT

STANDARD PROTOCOL ITEMS: RECOMMENDATIONS FOR INTERVENTIONAL TRIALS

SPIRIT 2013 Checklist: Recommended items to address in a clinical trial protocol and related documents*

Section/item	Item No	Description	Addressed on page number
**Administrative information**			
Title	1	Descriptive title identifying the study design, population, interventions, and, if applicable, trial acronym	________1____
Trial registration	2a	Trial identifier and registry name. If not yet registered, name of intended registry	________3____
	2b	All items from the World Health Organization Trial Registration Data Set	_______N/A___
Protocol version	3	Date and version identifier	_______22____
Funding	4	Sources and types of financial, material, and other support	_______28____
Roles and responsibilities	5a	Names, affiliations, and roles of protocol contributors	________1____
	5b	Name and contact information for the trial sponsor	________9____
	5c	Role of study sponsor and funders, if any, in study design; collection, management, analysis, and interpretation of data; writing of the report; and the decision to submit the report for publication, including whether they will have ultimate authority over any of these activities	________28____
	5d	Composition, roles, and responsibilities of the coordinating centre, steering committee, endpoint adjudication committee, data management team, and other individuals or groups overseeing the trial, if applicable (see Item 21a for data monitoring committee)	_________21____

For peer review only

**Introduction**			
Background and rationale	6a	Description of research question and justification for undertaking the trial, including summary of relevant studies (published and unpublished) examining benefits and harms for each intervention	_____5-7_____
	6b	Explanation for choice of comparators	______6______
Objectives	7	Specific objectives or hypotheses	________8_____
Trial design	8	Description of trial design including type of trial (eg, parallel group, crossover, factorial, single group), allocation ratio, and framework (eg, superiority, equivalence, noninferiority, exploratory)	________8_____
**Methods: Participants, interventions, and outcomes**			
Study setting	9	Description of study settings (eg, community clinic, academic hospital) and list of countries where data will be collected. Reference to where list of study sites can be obtained	_________9____
Eligibility criteria	10	Inclusion and exclusion criteria for participants. If applicable, eligibility criteria for study centres and individuals who will perform the interventions (eg, surgeons, psychotherapists)	________10____
Interventions	11a	Interventions for each group with sufficient detail to allow replication, including how and when they will be administered	________14____
	11b	Criteria for discontinuing or modifying allocated interventions for a given trial participant (eg, drug dose change in response to harms, participant request, or improving/worsening disease)	________19____
	11c	Strategies to improve adherence to intervention protocols, and any procedures for monitoring adherence (eg, drug tablet return, laboratory tests)	______19_____
	11d	Relevant concomitant care and interventions that are permitted or prohibited during the trial	________12____
Outcomes	12	Primary, secondary, and other outcomes, including the specific measurement variable (eg, systolic blood pressure), analysis metric (eg, change from baseline, final value, time to event), method of aggregation (eg, median, proportion), and time point for each outcome. Explanation of the clinical relevance of chosen efficacy and harm outcomes is strongly recommended	________16____
Participant timeline	13	Time schedule of enrolment, interventions (including any run-ins and washouts), assessments, and visits for participants. A schematic diagram is highly recommended (see Figure)	______Figure 1__

For peer review only - http://bmjopen.bmj.com/site/about/guidelines.xhtml

Sample size	14	Estimated number of participants needed to achieve study objectives and how it was determined, including clinical and statistical assumptions supporting any sample size calculations	______18_____
Recruitment	15	Strategies for achieving adequate participant enrolment to reach target sample size	______18_____

Methods: Assignment of interventions (for controlled trials)

Allocation:			
Sequence generation	16a	Method of generating the allocation sequence (eg, computer-generated random numbers), and list of any factors for stratification. To reduce predictability of a random sequence, details of any planned restriction (eg, blocking) should be provided in a separate document that is unavailable to those who enrol participants or assign interventions	______14_____
Allocation concealment mechanism	16b	Mechanism of implementing the allocation sequence (eg, central telephone; sequentially numbered, opaque, sealed envelopes), describing any steps to conceal the sequence until interventions are assigned	_______14____
Implementation	16c	Who will generate the allocation sequence, who will enrol participants, and who will assign participants to interventions	______14_____
Blinding (masking)	17a	Who will be blinded after assignment to interventions (eg, trial participants, care providers, outcome assessors, data analysts), and how	______8_____
	17b	If blinded, circumstances under which unblinding is permissible, and procedure for revealing a participant's allocated intervention during the trial	_______N/A____

Methods: Data collection, management, and analysis

Data collection methods	18a	Plans for assessment and collection of outcome, baseline, and other trial data, including any related processes to promote data quality (eg, duplicate measurements, training of assessors) and a description of study instruments (eg, questionnaires, laboratory tests) along with their reliability and validity, if known. Reference to where data collection forms can be found, if not in the protocol	_____15______
	18b	Plans to promote participant retention and complete follow-up, including list of any outcome data to be collected for participants who discontinue or deviate from intervention protocols	______15,19____

For peer review only - http://bmjopen.bmj.com/site/about/guidelines.xhtml

Data management	19	Plans for data entry, coding, security, and storage, including any related processes to promote data quality (eg, double data entry; range checks for data values). Reference to where details of data management procedures can be found, if not in the protocol	______21_____
Statistical methods	20a	Statistical methods for analysing primary and secondary outcomes. Reference to where other details of the statistical analysis plan can be found, if not in the protocol	_____18______
	20b	Methods for any additional analyses (eg, subgroup and adjusted analyses)	______18_____
	20c	Definition of analysis population relating to protocol non-adherence (eg, as randomised analysis), and any statistical methods to handle missing data (eg, multiple imputation)	______18_____
**Methods: Monitoring**			
Data monitoring	21a	Composition of data monitoring committee (DMC); summary of its role and reporting structure; statement of whether it is independent from the sponsor and competing interests; and reference to where further details about its charter can be found, if not in the protocol. Alternatively, an explanation of why a DMC is not needed	_______21______
	21b	Description of any interim analyses and stopping guidelines, including who will have access to these interim results and make the final decision to terminate the trial	______19_______
Harms	22	Plans for collecting, assessing, reporting, and managing solicited and spontaneously reported adverse events and other unintended effects of trial interventions or trial conduct	_______21_____
Auditing	23	Frequency and procedures for auditing trial conduct, if any, and whether the process will be independent from investigators and the sponsor	______21_____
**Ethics and dissemination**			
Research ethics approval	24	Plans for seeking research ethics committee/institutional review board (REC/IRB) approval	______22_____
Protocol amendments	25	Plans for communicating important protocol modifications (eg, changes to eligibility criteria, outcomes, analyses) to relevant parties (eg, investigators, REC/IRBs, trial participants, trial registries, journals, regulators)	_______22_____

For peer review only - http://bmjopen.bmj.com/site/about/guidelines.xhtml

Consent or assent	26a	Who will obtain informed consent or assent from potential trial participants or authorised surrogates, and how (see Item 32)	_______11______
	26b	Additional consent provisions for collection and use of participant data and biological specimens in ancillary studies, if applicable	_________N/A___
Confidentiality	27	How personal information about potential and enrolled participants will be collected, shared, and maintained in order to protect confidentiality before, during, and after the trial	________11/22___
Declaration of interests	28	Financial and other competing interests for principal investigators for the overall trial and each study site	________29____
Access to data	29	Statement of who will have access to the final trial dataset, and disclosure of contractual agreements that limit such access for investigators	_______22______
Ancillary and post-trial care	30	Provisions, if any, for ancillary and post-trial care, and for compensation to those who suffer harm from trial participation	______N/A_______
Dissemination policy	31a	Plans for investigators and sponsor to communicate trial results to participants, healthcare professionals, the public, and other relevant groups (eg, via publication, reporting in results databases, or other data sharing arrangements), including any publication restrictions	________22______
	31b	Authorship eligibility guidelines and any intended use of professional writers	_________22___
	31c	Plans, if any, for granting public access to the full protocol, participant-level dataset, and statistical code	_________N/A___
**Appendices**			
Informed consent materials	32	Model consent form and other related documentation given to participants and authorised surrogates	______supplied_
Biological specimens	33	Plans for collection, laboratory evaluation, and storage of biological specimens for genetic or molecular analysis in the current trial and for future use in ancillary studies, if applicable	________N/A____

*It is strongly recommended that this checklist be read in conjunction with the SPIRIT 2013 Explanation & Elaboration for important clarification on the items. Amendments to the protocol should be tracked and dated. The SPIRIT checklist is copyrighted by the SPIRIT Group under the Creative Commons "Attribution-NonCommercial-NoDerivs 3.0 Unported" license.

For peer review only - http://bmjopen.bmj.com/site/about/guidelines.xhtml
