# Supplementary material for: Randomised controlled trial of the Limit of Detection of Troponin and ECG Discharge (LoDED) strategy versus usual care in adult patients with chest pain attending the emergency department: study protocol
Source: BMJ Open. 2018 Oct 2;8(10):e025339. doi: 10.1136/bmjopen-2018-025339 (PMC6169748; doi:10.1136/bmjopen-2018-025339)
Supplement: Reviewer comments [file bmjopen-2018-025339.reviewer_comments.pdf]

PEER REVIEW HISTORY

BMJ Open publishes all reviews undertaken for accepted manuscripts. Reviewers are asked to complete a checklist review form (**http://bmjopen.bmj.com/site/about/resources/checklist.pdf**) and are provided with free text boxes to elaborate on their assessment. These free text comments are reproduced below.

ARTICLE DETAILS

**TITLE (PROVISIONAL)**	Randomised controlled trial of the LoDED (Limit of Detection of Troponin and ECG Discharge) strategy versus usual care in adult chest pain patients attending the Emergency Department: study protocol
**AUTHORS**	Carlton, Edward; Campbell, Sarah; Ingram, Jenny; Kandiyali, Rebecca; Taylor, Hazel; Aziz, Shahid; Beresford, Peter; Kendall, Jason; Reuben, Adam; Smith, Jason; Vickery, Patricia; Benger, Jonathan

This article was not externally reviewed at BMJ Open. Protocols that have been independently assessed prior to submission to BMJ Open are usually fast-tracked to publication on the grounds that further substantial changes will not be possible. This independent assessment will usually be external, independent review for both a substantial grant award from a non-commercial or government funder and ethics approval.
